# Do Readers Integrate Phonological Codes Across Saccades? A Bayesian Meta-Analysis and a Survey of the Unpublished Literature

**DOI:** 10.5334/joc.87

**Published:** 2019-10-04

**Authors:** Martin R. Vasilev, Mark Yates, Timothy J. Slattery

**Affiliations:** 1 Bournemouth University, Department of Psychology, GB; 2 University of South Alabama, Department of Psychology, US

**Keywords:** Reading, Eye movements, Attention, Vision, Visual word processing

## Abstract

It is commonly accepted that phonological codes can be activated parafoveally during reading and later used to aid foveal word recognition- a finding known as the phonological preview benefit. However, a closer look at the literature shows that this effect may be less consistent than what is sometimes believed. To determine the extent to which phonology is processed parafoveally, a Bayesian meta-analysis of 27 experiments and a survey of the unpublished literature were conducted. While the results were generally consistent with the phonological preview benefit (>90% probability of a true effect in gaze durations), the size of the effect was small. Readers of alphabetical orthographies obtained a modest benefit of only 4 ms in gaze durations. Interestingly, Chinese readers showed a larger effect (6–14 ms in size). There was no difference in the magnitude of the phonological preview benefit between homophone and pseudo-homophone previews, thus suggesting that the modest processing advantage is indeed related to the activation of phonological codes from the parafoveal word. Simulations revealed that the results are relatively robust to missing studies, although the effects may be 19–22% smaller if all missing studies found a null effect. The results suggest that while phonology can be processed parafoveally, this happens only to a limited extent. Because phonological priming effects in single-word recognition are small (10–13 ms; Rastle & Brysbaert, 2006) and there is a loss of visual acuity in the parafovea, it is argued that large phonological preview benefit effects may be unlikely.

Skilled adult reading involves the complex coordination of oculomotor and cognitive control that guides the readers’ eyes across the page. Because visual acuity decreases rapidly away from the centre of vision (the *fovea*), readers need to fixate individual words in the text in order to bring them into focus for processing. While this is crucial for word recognition, there is now a lot of evidence indicating that readers can also process words in the *parafovea*, or the area of the visual field that extends 2 to 5° from the point of fixation (Rayner, 1998). When readers have a valid preview of the upcoming word in parafoveal vision, they are faster to recognise it once this word is fixated compared to when valid preview is not available. This finding is known as the *preview benefit* effect (Rayner, 1998, 2009; for a review, see Schotter, Angele, & Rayner, 2012). This benefit is assumed to reflect a processing advantage that gives readers a head start in recognising the upcoming word and allows them to meet the neurological and oculomotor constraints imposed by saccadic eye-movements (Rayner & Reingold, 2015; Reichle & Reingold, 2013).

The preview benefit is arguably one of the most robust and consistently replicated effects in the literature of eye-movements during reading. However, one long-standing question has been what type of linguistic information can be acquired from parafoveal vision. This is crucial in understanding the role of parafoveal processing during reading because readers typically need to obtain different types of linguistic information to process the text- such as orthography, phonology, morphology, semantics, and syntactic information. While phonology is known to play a role in single-word visual recognition (e.g., Rastle & Brysbaert, 2006), the evidence in support of parafoveal processing of phonology has not been systematically assessed until now. In the present study, we report a Bayesian meta-analysis and a survey of the unpublished literature that investigated whether phonological information is integrated across saccades.

While a few selective reviews have at least briefly considered the parafoveal processing of phonology (Cutter, Drieghe, & Liversedge, 2015; Leinenger, 2014; Milledge & Blythe, 2019; Schotter et al., 2012), none of them has done a systematic search of the literature or tried to synthesize all the available evidence. This is important as each study has its own sampling and measurement error that can influence the estimation of the population effect. The evidence for an effect in Psychology is typically based on *p*-values. However, *p*-values have a considerable sample-to-sample variability for all but the most statistically powerful designs (Halsey, Curran-everett, Vowler, & Drummond, 2015). Because psychological experiments often have low statistical power (Vankov, Bowers, & Munafò, 2014), this can lead to spurious conclusions when comparing results across studies based on statistical significance (Gelman & Stern, 2006). Additionally, no studies to date have explicitly considered the existence of unpublished studies on the topic. This is important as the preferential publishing of studies that “worked” (i.e., the ones with *p*-values <0.05) can lead to overoptimistic expectations of replicability (Vasishth, Mertzen, Jäger, & Gelman, 2018). Therefore, the present study aimed to synthesize all the available evidence to: 1) find out the probability that readers process phonology parafoveally; and 2) estimate the size of the effect and the uncertainty around it.

## The Preview Benefit during Reading

**Figure 1:**
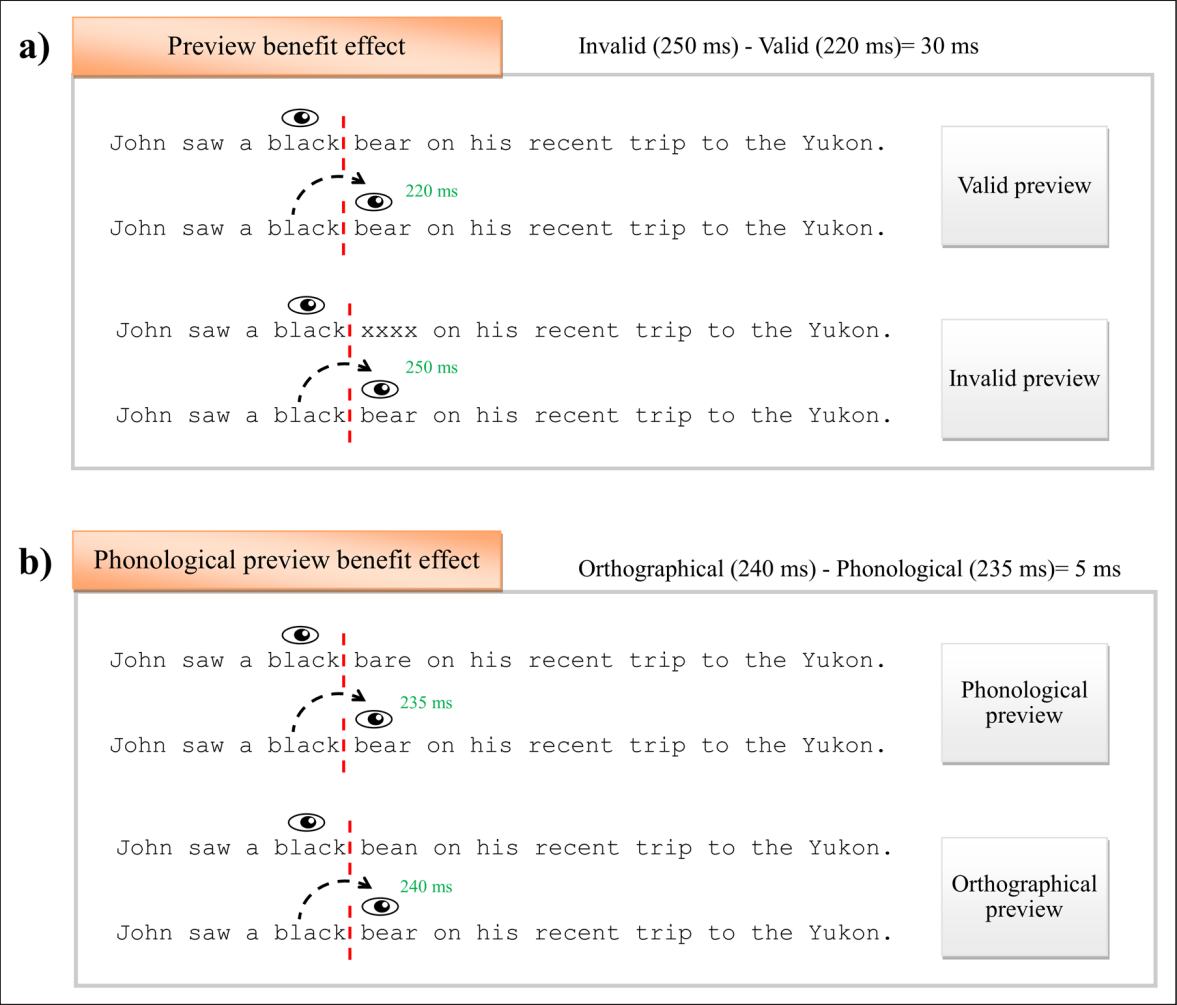
An illustration of the preview benefit **(a)** and phonological preview benefit **(b)** in English using the boundary technique (Rayner, 1975). The invisible boundary is illustrated with vertical dotted lines. In Chinese studies, the baseline for the phonological preview benefit effect is typically an unrelated-word preview (see the main text). In the classical preview benefit effect (panel a), different invalid preview masks can be used, e.g.: bear -> hurm (pseudoword), bear -> txvu (random letter mask), or bear -> tuna (unrelated word).

The preview benefit (PB) effect is typically investigated with the boundary paradigm (Rayner, 1975). In this technique, an invisible boundary is placed immediately before a target word in the sentence. Once participant’s gaze moves to the right of the invisible boundary, the preview changes to the actual target word. The classical PB effect refers to the finding that fixation durations are shorter when participants have a valid preview of the target word (e.g., “bear”) compared to when such preview is denied (e.g., the word “bear” is masked by a string of Xs). This is illustrated in Figure 1. The PB effect is usually about 25–45 ms, although its exact size depends on the measure and the type of invalid preview baseline that is used (Vasilev & Angele, 2017).

The advantage of the boundary paradigm is that it makes it possible to precisely manipulate what linguistic information is available in parafoveal vision while readers are still fixating on the previous word. This has led to a large body of evidence investigating whether readers can process orthographic (Balota, Pollatsek, & Rayner, 1985; Johnson, Perea, & Rayner, 2007; Snell, Vitu, & Grainger, 2017; Williams, Perea, Pollatsek, & Rayner, 2006), phonological (Chace, Rayner, & Well, 2005; Miellet & Sparrow, 2004; Pan, Laubrock, & Yan, 2016; Pollatsek, Lesch, Morris, & Rayner, 1992), semantic (Hohenstein & Kliegl, 2014; Rayner, Balota, & Pollatsek, 1986; Rayner, Schotter, & Drieghe, 2014; Schotter, 2013; Veldre & Andrews, 2016a; Yan, Richter, Shu, & Kliegl, 2009), morphological (Deutsch, Frost, Pelleg, Pollatsek, & Rayner, 2003; Deutsch, Frost, Pollatsek, & Rayner, 2005; Kambe, 2004; Stoops & Christianson, 2017), and even syntactic information in parafoveal vision (Brothers & Traxler, 2016; Kim, Radach, & Vorstius, 2011; Snell, Meeter, & Grainger, 2017; Veldre & Andrews, 2018). Importantly for the present research, there seems to be a consensus in the literature that orthographic and phonological information is routinely integrated across fixations (Cutter et al., 2015; Schotter et al., 2012).

## Computational Models of Eye-movement Control during Reading

There are now a number of relatively successful models of eye-movement control during reading (Engbert, Longtin, & Kliegl, 2002; Engbert, Nuthmann, Richter, & Kliegl, 2005; Pollatsek, Reichle, & Rayner, 2006; Reichle, Pollatsek, Fisher, & Rayner, 1998; Reichle, Rayner, & Pollatsek, 2003; Reichle, Warren, & McConnell, 2009; Reilly & Radach, 2003, 2006; Risse, Hohenstein, Kliegl, & Engbert, 2014; Schad & Engbert, 2012; Seelig et al., 2019; Snell, van Leipsig, Grainger, & Meeter, 2018). All of these models are capable of capturing important aspects of oculomotor behaviour during reading, such as fixation locations within words, refixation rates, and fixation durations. Furthermore, all models assume that the processing of upcoming words in parafoveal vision plays an important role in fluent reading. While these models differ substantially in how they implement parafoveal processing, they are all capable of accounting for the PB effects that would derive from boundary change experiments with invalid previews, as illustrated in Figure 1a. The PB within E-Z Reader (Reichle et al., 1998) results from a decoupling of attention for word processing and oculomotor programming. Attention arrives at a word in advance of an eye fixation which allows for lexical processing. However, E-Z Reader is agnostic as to the specific codes involved in lexical processing. Instead, lexical processing is simply a function of a word’s frequency and its predictability.

While SWIFT (Engbert et al., 2005) differs considerably from E-Z Reader in how attention is allocated to words for lexical processing, it uses a similar shortcut where the word activations involved in lexical processing are functions of a word’s frequency and predictability. The most recent model, OB1 Reader (Snell et al., 2018), does implement word recognition through the use of an open bigram coding scheme in which a word will activate all the relative position bigrams it contains. So, *word* would activate *wo, wr, wd, or, od*, and *rd*. This allows OB1 Reader to make more specific predictions about PB effects than any of its predecessors. Furthermore, looking at the example in Figure 1b, we can see that the phonological preview *bare* (***ba, br, be, ar**, ae, re*) shares 4 of 6 open bigrams with the target *bear* (be, ba, br, ea, er, ar) while the orthographic control *bean* (***be, ba,** bn, **ea,** en, an*) only shares 3 of 6 open bigrams with it. Therefore, even though OB1 Reader does not explicitly code phonological representations, it may be capable of predicting a phonological PB effect based on the correlation between shared open bigrams and shared phonological codes. Still, the phonological PB effect (i.e., orthographic – phonological preview) in this example would be 1/6 the size of the PB effect with an invalid preview which shares zero open bigrams with the target. Therefore, measurement variance may have a large influence on reliably detecting the phonological PB effect. This highlights the need for a clear understanding of the size of the phonological PB effect for future model development and selection.

## Phonological Processing in Single Word Recognition

While models of oculomotor control have been successful at reproducing fixation patterns during reading, models of single-word recognition are far more specific about the details of lexical processing and the role that phonological codes play in it. There are two general accounts of phonological processing in visual word recognition. According to the strong view (Frost, 1998), the rapid computation of a phonological code is a mandatory component of visual word recognition. In contrast, according to the weak phonological view (Rastle & Brysbaert, 2006), word recognition can occur both through a direct orthographic pathway and through an indirect pathway that is mediated by phonology. The weaker view has been implemented in models such as the dual-route cascaded model (DRC; Coltheart, Rastle, Perry, Langdon, & Ziegler, 2001) and the connectionist-dual process model (CDP+, Perry, Ziegler, & Zorzi, 2007), both of which include a lexical pathway where the orthographic word form can be activated directly from the incoming letter-level activation. While both models also contain a sub-lexical pathway, they differ in how this is implemented: in the DRC, the grapheme-to-phoneme mapping is hardwired into the model, whereas in the CDP+ the sublexical pathway consists of a connectionist network that learns by exposure to the language. Nevertheless, both models assume that the role of sublexical activation on the orthographic word form is indirect. This is contrary to the strong view, which posits that sublexical phonological activation is the foundation on which visual word recognition proceeds.

One way to adjudicate between the strong and weak accounts is by assessing the reliability of masked phonological priming where target words are responded to more rapidly and/or accurately when followed by a phonologically similar prime (e.g., *hirt*) than a phonologically dissimilar orthographic control (e.g., *hort*). According to the strong view, the phonological code is rapidly computed from the orthographic input, and as such, masked phonological priming should exist. In fact, some have argued that the existence of masked phonological priming is inconsistent with the weak view and that only the strong phonological view can accommodate the effect (Frost, 1998; Lukatela & Turvey, 1994a, 1994b). The problem, however, is that the masked phonological priming effect has been called into question, with some even doubting the effect is real. This led Rastle and Brysbaert (2006) to conduct an exhaustive meta-analysis. They concluded that masked phonological priming does exist in visual word recognition, although the size of the effect can vary as a function of the masking paradigm.

In visual word recognition, three types of responses have been used to evaluate masked phonological priming. Some of the earliest work used perceptual identification with either forward masking (pattern mask -> briefly presented word/nonword prime -> briefly presented target -> pattern mask) or backward masking (pattern mask -> briefly presented target word -> briefly presented word/nonword mask -> pattern mask). The participants’ task is to correctly identify the target word by writing or typing the word. However, backward masking has been seriously criticized as favouring phonological processing because the visual masking from the backward mask is highly disruptive to the orthographic processing of the target word (Verstaen, Humphreys, & Olson, 1995). Today, masked phonological priming is more commonly studied with either forward-masked speeded naming (pattern mask -> briefly presented word/nonword prime -> target word until read aloud) or forward-masked lexical decision (pattern mask -> briefly presented word/nonword prime -> letter string requiring lexical decision). In these paradigms, the target stimulus occurs immediately after the prime and thus acts to mask it so that participants are unaware of the prime.

Rastle and Brysbaert (2006) found the largest effect size in the forward-masked reading aloud paradigm (mean effect size *r* = .312). This is hardly surprising as the reading aloud task requires computation of a phonological code, while lexical decisions can be, in principle, performed correctly by relying purely on an orthographic recognition process. Nevertheless, they did find mean effect sizes indicating a small to medium effect in each of the other masked priming paradigms (mean effect size *r* ranged from .193–.240). Taken together, their results indicate that the phonological masked priming effect is real. As further evidence, they conducted two new forward-masked lexical decision experiments that revealed a reliable, small masked phonological priming effect. Overall, the phonological priming effects measured in milliseconds in the meta-analysis and their own experiments ranged from 9 to 13 ms. In simulation work, they found that the DRC model was unable to simulate the masked phonological priming effect if the lexical decision was made based on orthographic activation within the orthographic lexicon. Making changes to the parameters of the model did allow it to come closer to simulating the effect, but at the cost that it could no longer read exception words. However, when they allowed the model to make the lexical decision based on phonological activation, rather than orthographic activation, it fared much better. It seems then that a model that holds to the weak phonological view can simulate the masked phonological priming effect if lexical decisions are made based on phonological rather than orthographic information, but this brings the model more in line with the strong phonological view. To be fair, there has never been a computational model that adheres to the strong phonological view, so it is unknown how it would fare in simulating not only masked phonological priming effects, but also other benchmark effects (e.g., frequency, neighbourhood effects).

## Phonological Processing in the Parafovea

### Alphabetical orthographies

Pollatsek et al. (1992) were first to show evidence for phonological PB during reading of English sentences. They found that parafoveal previews that are homophonous with the target word (e.g. “bare” as the preview of “bear”) led to significantly shorter first fixation durations compared to an orthographic control condition (e.g., “bean” as the preview of “bear”). This effect was 20 ms in size and was taken as evidence that the phonological information from the parafovea is preserved across fixations in order to aid foveal word recognition. Further support for this conclusion was found by Miellet and Sparrow (2004), who reported that French pseudo-homophone previews (e.g., “cheise” as the preview of “chaise”) also resulted in shorter fixation durations compared to visually similar pseudo-word control previews (e.g., “choise” as the preview of “chaise”). While explicit statistical comparisons between pseudo-homophone and pseudo-control previews were not reported, the condition means were consistent with the pattern of results obtained by Pollatsek et al. (1992). Because pseudo-homophones do not exist in participants’ phonological lexicon, Miellet and Sparrow (2004) interpreted their findings as evidence that phonological codes are assembled parafoveally before the target word is fixated.

Chace et al. (2005) further investigated how reading skill may modulate the effect. They found that if they excluded participants who scored lower than the 20-percentile rank on the Nelson-Denny Reading Test, there was evidence of a significant phonological PB effect in gaze durations. The authors interpreted this as a replication of Pollatsek et al. (1992) and motivated the exclusion of low-scoring participants by arguing that “…such a high concentration of low-level readers is not indicative of the general student population (Chace et al., 2005, p. 211)”. Additionally, when only data from the homophone and orthographic control conditions were considered, “more skilled” readers (≥60 percentile) showed a robust phonological PB effect in gaze durations (a mean difference of 35 ms), whereas “less-skilled” readers (≤40 percentile) showed no evidence for phonological PB (a difference of –15 ms). Chace et al.’s results suggest that only skilled adult readers benefit from phonological information in the parafovea. However, another interpretation may be that poor readers add noise to the data, which makes it difficult to detect small effects. Because no theory to our knowledge would predict a negative difference instead of a zero difference in the less-skilled group, this could indicate that the study had non-negligible measurement error.

Bélanger, Mayberry, and Rayner (2013) investigated the effect in skilled and less-skill deaf readers, as well as in a control group of hearing adults. While neither the skilled nor the less-skilled deaf readers showed evidence for phonological PB, the hearing controls did show evidence for the effect in first fixation duration (24 ms). However, this effect was only apparent when the phonological preview word was high frequency and the target word was low frequency. Bélanger et al. (2013) also considered this to be a replication of previous findings (Chace et al., 2005; Miellet & Sparrow, 2004; Pollatsek et al., 1992), arguing that the frequency of parafoveal words may influence the activation of letter identities, and therefore the processing of phonological information in the parafovea. Interestingly, however, most target/homophone preview pairs used in their study were borrowed from Pollatsek et al. (1992), who reported a general phonological PB effect irrespective of lexical frequency. Another experiment by Choi and Gordon (2014), which also partly borrowed from Pollatsek et al.’s (1992) targets, found no evidence for phonological PB with either homophone or pseudo-homophone previews.

Tiffin-Richards and Schroeder (2015) investigated the parafoveal processing of phonology in child and adult German readers by comparing pseudo-homophones (e.g., Bläch- Blech) to orthographic controls (e.g., Blüch- Blech). While children showed phonological PB effects in single fixation duration (28 ms) and gaze duration (34 ms), the adults did not. Tiffin-Richards and Schroeder (2015) argued that the lack of an effect in adults may be due to the greater orthographic transparency of German and the fact that their reading stimuli were optimized for children. More recently, Blythe, Dickins, Kennedy, and Liversedge (2018) tested the parafoveal processing of phonology in English teenagers with permanent hearing loss, as well as in reading- and age-level hearing controls. Interestingly, all three groups showed a significant phonological PB effect with peseudo-homophone previews in gaze durations, thus suggesting that they were able to activate the phonological codes of the parafoveal word.

To examine whether the phonological PB can still be observed even in the absense of orthographic similarity, Jouravlev and Jared (2018) tested English-Russian bilinguals. They presented Russian preview words written in the Cyrilic alphabet that had phonological but little or no othographic overlap with the English target words (e.g., “бланк [blank]– blood”) and compared them to previews that had neither phonological nor orthographic overlap (e.g., “гжель [ɡʐɛlʲ]–blood”). Jouravlev and Jared (2018) reported a significant phonological PB effect independent of orthography for both first fixation duration (20 ms) and gaze duration (43 ms). More recently, Leinenger (2018) also examined the phonological PB in English readers. She found significant evidence for phonological PB in English with pseudo-homophone previews (Experiment 4), but not with homophones previews (Experiment 3). In both experiments, the size of the effect was comparable, although it was considerably more modest than that of previous studies (6–8 ms).

To summarise, classical studies have shown that readers of alphabetical orthographies can process phonological information parafoveally (Miellet & Sparrow, 2004; Pollatsek et al., 1992). However, a closer look at the literature shows that this effect may be less consistent than what is sometimes believed. While some recent studies have replicated the effect (Blythe et al., 2018; Leinenger, 2018, Experiment 4), others have either not replicated it (Choi & Gordon, 2014; Leinenger, 2018, Experiment 3) or have found it only after data exclusions based on factors, such as the reading skill of participants (Chace et al., 2005) or the lexical frequency of the preview and the target word (Bélanger et al., 2013). While not explicitly stated, some of the modulation analyses could have been done post-hoc, as they were not always incorporated in the study design. This raises questions about the generalisability of the effect across different populations and reading situations.

It is also interesting to note that some of the early studies have found phonological PB effects that are similar in size to the classical PB effect (valid vs invalid preview difference; see Figure 1a for an illustration), which has been estimated to be 26 ms on average for first fixation duration and 37 ms for gaze durations (Vasilev & Angele, 2017). As the source of the classical PB effect is assumed to be some combination of abstract letter and phonological codes (Rayner, White, Kambe, Miller, & Liversedge, 2003), this raises the possibility that these early studies may have overestimated the size of the phonological PB effect. Estimating the exact size of the effect is important for designing experiments that are sufficiently powered to detect it.

### Mandarin Chinese

Most studies on the phonological PB have been conducted in alphabetical orthographies such as English. However, as phonology is not always represented in the same way across different languages, there could be cross-linguistic differences in the effect. For example, languages with non-alphabetical orthographies such as Mandarin Chinese differ from English in a number of ways that could potentially influence the parafoveal processing of phonology (see Tsang & Chen, 2012; Yu & Reichle, 2017). Unlike English, Chinese uses a logographic writing system where characters, rather than letters, are the basic unit. Chinese is an unspaced language because individual characters are not demarcated by spaces and there are no boundaries between words. Most words in Chinese are one or two characters long, although some words can consist of four or more characters (X. Li, Zang, Liversedge, & Pollatsek, 2015). Chinese characters are square-shaped and are made up of basic strokes (Wang & Yang, 2007). Some of the strokes can be combined to form radicals and each character consists of at least one such radical.

Around 80% of all Chinese characters are compound characters, which are made up of a phonological and one or more semantic radicals (Chen, 1996; Shu, 2003). The semantic radical is usually located on the left side of the character and the phonological one is located on the right side of the character, although this is not a fixed feature of the language and their positions can vary (Chen, 1996). While the semantic radical provides information about the meaning of the character, the phonological one provides a guide to its pronunciation, though not always a very reliable one (Shu, 2003). This is because phonological information is more loosely encoded in Chinese characters due to the very long history of the language during which multiple phonologies of individual character components have emerged (Honorof & Feldman, 2004). For example, Wengang (1991) took all compound characters from a dictionary and calculated that the average reliability with which phonetic radicals represent the pronunciation of their characters is only 0.58. This is the case because only 36% of phonetical radicals correctly represent the pronunciation of compound characters, 48% partially represent the pronunciation, and 16% do not represent the pronunciation of the character at all (Wengang, 1991). Therefore, phonetic radicals do not consistently provide reliable information regarding the pronunciation of such compound characters.

Liu et al. (2002) were first to investigate the parafoveal processing of phonology during the reading of Chinese. They reported that a phonologically similar character preview (e.g., 药 as the preview of 要) led to significantly shorter gaze durations and total viewing time compared to an unrelated preview character that was neither phonologically nor orthographically similar to the target (e.g., 位 as the preview of 要). However, this result was significant only when the authors analysed fixation durations on a target area that included the target, pre-target, and post-target character. Tsai et al. (2004) replicated the phonological PB effect in gaze durations and further extended Liu et al.’s (2002) results by showing that visually similar homophones also lead to a significant phonological PB in first fixation duration. Tsai et al. (2004) also found that characters with highly consistent phonology (i.e., the ones where the phonetic radical consistently represents the same pronunciation across different characters) showed phonological PB in first fixation duration, whereas those with lower consistency did not. The authors argued that this last result suggests that Chinese readers can access the phonological information from parafoveal vision and use it in the early stages of character identification.

More recently, Yan et al. (2009) found a numerical trend that was consistent with the phonological PB effect in gaze durations with simple Chinese characters, but the effect did not reach significance. Interestingly, Tsai, Kliegl, and Yan (2012) adapted Yan et al.‘s (2009) stimuli for readers in Taiwan (where traditional Chinese characters are used), but failed to find a significant phonological PB or any indication of an effect in that direction. Nevertheless, post-hoc analyses indicated that the effect became significant with increasing preview time (i.e., longer gaze durations on the pre-target word), but only when that word was of high frequency. These early results suggest that, while the effect may exist in Mandarin Chinese, it may be weaker under certain circumstances.

Pan et al. (2016) further investigated how reading mode (silent vs oral) may modulate the effect. Because oral reading requires the articulation of words, Pan et al. predicted that the phonological PB should be larger in oral compared to silent reading due to the greater relevance of phonological information in the former mode. Contrary to this prediction, Experiment 1 found a significant phonological PB in silent, but not in oral reading, using the same simple character previews from Yan et al.‘s (2009) study. Nevertheless, as there was a significant phonological parafoveal-on-foveal effect in oral reading, the authors argued that the results are still consistent with the notion that phonological processing is greater during oral reading. Additionally, Experiment 2 provided direct evidence for this hypothesis using compound characters, as the phonological PB was significant for both first fixation duration and gaze duration in oral reading, but not in silent reading (although there was a numerical trend in second-pass measures). Therefore, these results suggest that there may be greater parafoveal processing of phonology during oral reading, at least for compound characters.

More recently, Luo, Wu, and Jiao (2018) investigated the role of phonetic radicals on the parafoveal processing of phonology. In line with the results of Tsai et al. (2004), they found that readers were able to process phonology at the radical level, which was shown by a significant difference between characters with regular phonology and characters with irregular phonology in first fixation durations. In this study, regularity referred to whether the compound character had the same pronunciation as its phonetic radical or not. Interestingly, however, no phonological PB was found at the character level, because a compound character that was homophonous with the target character but did not share the same phonetic radical did not differ from an unrelated character. This last result is contrary to some previous findings (e.g., Liu et al., 2002; Pan et al., 2016) and further highlights the need for a systematic analysis of the phonological PB effect.

In summary, despite the differences with which phonology is represented in Chinese, there is some evidence indicating that readers may be able to activate phonological information parafoveally (Liu et al., 2002; Luo et al., 2018; Pan et al., 2016; Tsai et al., 2004). Nevertheless, there are also some inconsistencies in the results. Future research may verify that the effect is moderated by additional factors, such as the type of characters used and/or the reading mode (Luo et al., 2018; Pan et al., 2016), or properties of the pre-target character and preview time (Tsai et al., 2012). However, we are interested in determining the size of the general effect (i.e., averaged over all moderating factors). As there are also a number of inconsistencies in the results from alphabetical orthographies, it is unclear whether the size of the phonological PB effect may differ between Mandarin and English.

## Present Study

There seems to be a general consensus in the literature that phonological codes are integrated across saccades during reading (Clifton Jr., 2015; Cutter et al., 2015; Leinenger, 2014; Milledge & Blythe, 2019; Schotter et al., 2012). While this conclusion may often be accepted as a fact, there has been no attempt to make a statistical synthesis of all the available evidence supporting the existence of this effect. Therefore, one of the theoretical contributions of this work was to test whether the effect is reliably bigger than 0. If the effect is not reliable, this would clearly be at odds with theories that assume its existence. Even though Vasilev and Angele (2017) analysed studies in the boundary paradigm, they did not consider the phonological PB effect. Moreover, it is currently not known how big the phonological PB effect is or how its magnitude compares to the phonological priming effects typically observed in the single word recognition literature. As phonological priming effects in English are typically around 10–13 ms (Rastle & Brysbaert, 2006), it could be speculated that the phonological PB effect may be even smaller in size. This is because the phonological prime in boundary experiments is never directly fixated and readers only glimpse it in the parafovea where visual acuity is more limited. The exact magnitude of the effect has theoretical implications for reading research because it can be used to constrain theories and computational models of reading. For example, any prospective model of reading that simulates phonological preview benefits that are considerably larger than our estimate would be at odds with the available evidence.

Furthermore, it is currently not known whether the measured effect size differs between alphabetical orthographies such as English and non-alphabetical orthographies such as Chinese. This is of interest as phonological information in Chinese does not follow the same spelling-to-sound mapping as alphabetical orthographies. While it has been sometimes argued that the phonological PB effect is less robust in Chinese compared to English (Tsang & Chen, 2012), this claim has not been empirically tested before. Finally, given the somewhat inconsistent results, it is also not clear how many participants and items may be needed to reliably detect the effect in both languages.

The main goal of the present study was to estimate the size of the phonological PB effect and the probability that it exists. To do so, a Bayesian meta-analysis was used (Schmid & Mengersen, 2013; Welton, Sutton, Cooper, & Ades, 2012). Historically, Bayesian approaches to meta-analysis have been more commonly used in the medical sciences (e.g., Higgins & Spiegelhalter, 2002; Sutton & Abrams, 2001), although more recently they have also gained popularity in psychology and linguistics (Jäger, Engelmann, & Vasishth, 2017; Marsman et al., 2017; Nicenboim, Roettger, & Vasishth, 2018; Rouder & Morey, 2011; Rouder, Morey, & Province, 2013; Vasishth, Chen, Li, & Guo, 2013). The advantage of using Bayesian inference for evidence synthesis is that it makes it possible to quantify the uncertainty around the effect of interest and to make direct probabilistic inferences about it (e.g., What is the probability, given the data, that the phonological PB is bigger than 0 ms?). A secondary goal of the meta-analysis was to use Bayesian meta-regression models to test how different factors such as the language of the study or the type of phonological preview may modulate the size of the effect. More specifically, a particular interest was to compare how the effect differs between alphabetical and Chinese studies, and between homophone and pseudo-homophone previews for alphabetical orthographies. The final goal was to do a statistical power analysis using estimates from the meta-analysis that will help future studies to reliably detect the effect.

Another contribution of the present meta-analysis was that it addressed more directly the issue of publication bias and the availability of unpublished data. Publication bias, also known as the “file drawer” problem (Rosenthal, 1979), refers to the phenomenon that non-significant or “negative” findings are less likely to be submitted for publication and ultimately be published in peer-reviewed journals (Franco, Malhotra, & Simonovits, 2014). The presence of publication bias is problematic for meta-analyses if the retrieved studies are not representative of all studies that have been completed on the topic or if they have some sort of systematic bias (Rothstein, Sutton, & Borenstein, 2005). While a number of very useful methods have been developed for the detection and correction of publication bias (for a review, see Jin, Zhou, & He, 2015), they all rely on different statistical assumptions of how publication bias may manifest itself. In reality, the true existence of publication bias or its exact nature are unknown. Therefore, in addition to using conventional statistical tests to detect publication bias, an anonymous survey was conducted with researchers in this area in order to find out the extent to which unpublished studies exist on this topic, and to locate information about as many of them as possible. The findings from the survey were then used in statistical simulations to investigate how the missing studies may influence the results.

## Method

### Literature Search

The search of the literature followed the PRISMA guidelines (Moher et al., 2009) and covered all articles published until December 21, 2018. This was done by searching Google Scholar and the Web of Science with the terms “parafoveal preview AND reading” and “phonological preview AND reading” (two separate searches). In addition to this, the reference list of screened articles and their citations in Google Scholar were checked for relevant studies. The reference lists of recent literature reviews on the topic were also checked (Cutter et al., 2015; Leinenger, 2014; Schotter et al., 2012).

**Figure 2:**
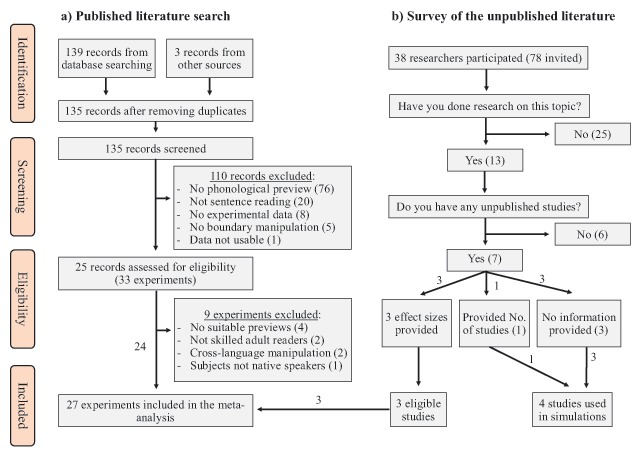
A flowchart of the literature search process **(a)** and results from the survey of the unpublished literature **(b)**. The data for one study could not be used because it included participants who had taken part in another included experiment. “Other sources” refers to studies located through methods other than database searching (e.g., contact with authors).

The study selection process is illustrated in Figure 2a. The abstracts of identified records were first screened for relevant studies and then any duplicates were removed. The full-texts were then screened for articles that did not fit the scope of the meta-analysis. Finally, the remaining articles were evaluated against the inclusion criteria presented in Appendix A. In short, the studies had to use the boundary paradigm (Rayner, 1975) to manipulate the phonological preview of a target word in skilled adult readers, have a suitable control condition and reasonable eye-tracking data quality. As a result of this evaluation, 24 studies were included in the meta-analysis (72.7% of all considered experiments). Three more eligible studies were located from the survey of the unpublished literature (see below), thus bringing the total number of included studies to 27. More information about the included studies is presented in Table 1 for alphabetical studies and in Table 2 for Chinese studies.

**Table 1:** Alphabetical Studies Included in the Meta-analysis.

Study	Language	N	Items/cond	Preview type	Stimuli	Effect size in ms (SE)	

						FFD	GD

Vasilev et al. (2019), Exp.1^†^	English	64	10	PSH	Own	–5 (8.6)	7 (11.7)
Vasilev et al. (2019), Exp.2^†^	English	64	10	PSH	Own	3 (8.6)	2 (11.6)
Barrington (2018)^†^	English	23	16	H	Chace 2005	–3 (16.5)	–2 (21.7)
Blythe et al. (2018)	English	23	8	PSH	Own	9 (10.5)	13.5 (14.9)
Leinenger (2018), Exp.3	English	48	56	H	Own	6 (9.7)	7 (12.4)
Leinenger (2018), Exp.4	English	48	60	PSH	Own	6 (9.1)	8 (11.3)
Drieghe et al. (2016)^†^	English	95	N/A	H	Chace 2005	–4 (3.3)	–3 (4.5)
Plummer (2015), Exp.1^†^	English	68	14	H	Own	N/A	7 (7.15)
Plummer (2015), Exp.2a^†^	English	51	18	PSH	Own	N/A	9.5 (6.3)
Plummer (2015), Exp.2b^†^	English	53	24	PSH	Own	N/A	6.5 (6.7)
Tiffin-Richards et al. (2015)	German	23	14	PSH	Own	–4 (6.7)	–2 (8.6)
Worf (2015)^†^	English	12	16	H	Chace 2005	2 (14)	5 (17.8)
Choi & Gordon (2014)	English	48	12	PSH	Pollatsek 1992/Lee 1999	–7 (4.7)	–6 (6.9)
Bélanger et al. (2013)	English	20	18	H	Pollatsek 1992	10.5 (16)	6 (20.7)
Winskel (2011)	Thai	36	8	H	Own	–5 (7.2)	–17 (12)
Chace et al. (2005)	English	23	16	H	Own	7 (5.2)	9 (7.6)
Murray & Flynn (2005)^†^	English	30	7	H	Own	–5 (15.2)	22 (22.1)
Miellet & Sparrow (2004)	French	13	20	PSH	Own	13 (15.5)	15 (19.3)
Pollatsek et al. (1992), Exp.2	English	40	12	H	Own	20 (8)	14 (11)

*Note*: Positive effects indicate evidence for phonological preview benefit. PSH: pseudo-homophone. H: homophone. SE: standard error of the mean difference. FFD: first fixation duration. GD: gaze duration. N: number of participants. Items/cond: number of items per condition. N/A: data not available. ^†^ Unpublished study.

**Table 2:** Chinese Studies Included in the Meta-analysis.

Study	N	Items/cond	Stimuli	Effect size in ms (SE)	

				FFD	GD

Luo et al. (2018), Exp.1	24	10	Own	12 (20.1)	1 (39)
Luo et al. (2018), Exp.2	32	10	Own	13 (19.2)	13 (30.3)
Pan et al. (2016), Exp.1	54	15	Yan et al. (2009)	12 (5.8)	33 (8.6)
Pan et al. (2016), Exp.2	56	15	Own	5 (6.3)	10 (8.6)
Tsai et al. (2012)	50	10	Yan et al. (2009)^†^	–2 (5.8)	–4 (9.1)
Yan et al. (2009)	48	10	Own	3 (9.7)	11 (19.7)
Tsai et al. (2004), Exp.1	20	24	Own	8 (7.4)	22 (13.1)
Liu et al. (2002), Exp.1	27	10	Own	N/A	16 (15.9)

*Note*: Positive effects indicate evidence for phonological preview benefit. SE: standard error of the mean difference. FFD: first fixation duration. GD: gaze duration. N: number of participants. Items/cond: number of items per condition. N/A: data not available. ^†^ Study stimuli were adapted for traditional Chinese readers.

### A Survey of the Unpublished Literature

To estimate the proportion of unpublished studies on the topic and to gather information about them, a survey was conducted that targeted researchers who have done relevant previous work. Seventy-eight researchers were identified based on their previous publication record and were sent an email invitation to participate in an anonymous survey. Every attempt was made to invite all relevant researchers who have worked on this or similar topics and to make the sample as internationally representative as possible. Participants were asked to indicate whether: 1) they have worked on this topic before and 2) have any unpublished studies. If they indicated that they have unpublished studies, participants were asked if they can provide more information- either details that will allow these studies to be included in the meta-analysis (e.g., descriptive statistics) or simply the number of unpublished studies that they have (to be used in the statistical simulations). Participants also had the option to provide no information at all. The survey was conducted between December 12, 2018 – January 31, 2019.

The results of the survey are presented visually in Figure 2b. The survey response rate was 48.7%. From the researchers who took part, 34% indicated that they have done previous research on the topic. Out of the researchers who had done relevant research, 53.8% indicated that they have some unpublished data (7 individuals). Three out of the seven researchers provided effect sizes for one unpublished study each, which made it possible to include three more experiments into the meta-analysis. To avoid duplicate entries, participants were asked if they were the lead researcher on the project, and all indicated that they were. These studies met the same inclusion criteria from the search of the published literature. From the remaining four researchers with unpublished data, one researcher indicated that they have one unpublished study but provided no effect sizes, and three researchers provided no information at all. In summary, the survey located three unpublished studies that were included in the meta-analysis and found evidence for at least four more missing studies for which no further information could be obtained. The missing studies were used in statistical simulations to evaluate the robustness of the main results.

### Effect Size Calculation

Two dependent measures were used in the present meta-analysis: *first fixation duration* (FFD) and *gaze duration* (GD). FFD is the first fixation on a word during first-pass reading, while GD is the sum of all fixations on a word until readers move on to another word. The size of the phonological PB effect was calculated in milliseconds for each study. This was done by subtracting fixation durations in the phonological preview condition from the control condition (orthographic preview for alphabetical studies and unrelated word preview for Chinese studies). Because Chinese has a deep orthography and a higher number of homophones compared to languages such as English (see Duanmu, 2006), it is easier to create phonological previews that have little orthographic overlap with the target word. As a result, an orthographic control condition is not necessary in this language. This is likely why researchers have generally opted for an unrelated-word control condition that is neither phonologically nor orthographically related to the target. Positive effect sizes indicate evidence for the phonological PB, whereas negative effect sizes indicate evidence against it.

The variance of effect sizes was calculated using Formulas 12.8–12.9 from Borenstein (2009). More specifically, the variance of each study (*V*) was calculated as:

1 V = \;\frac{{S_{diff}^2}}{n}

where *S_diff_* is the standard deviation of the mean difference for that study and *n* is its sample size. *S_diff_* was calculated as:

2 {S_{diff}} = \;\sqrt {S_1^2 + S_2^2 - 2\; \times \;r\; \times \;{S_1} \times \;{S_2}\;}

where *S_1_* is the standard deviation of the phonological preview condition, *S_2_* is the standard deviation of the control condition, and *r* is the correlation between the means in the phonological and the control preview conditions. Because *r* could not be calculated for all studies, the population correlation between the phonological and the control condition (ρ) was estimated from six experiments for which the raw data was available. The six correlations were then meta-analysed to obtain a weighted estimate of ρ (Szalma & Hancock, 2011). The resulting estimate of ρ (0.64 for FFD and 0.62 for GD) was used for calculating the variances in all studies.

### Publication Bias

In the present meta-analysis, 33.3% of all included studies were not published. To assess the data for publication bias, funnel plots were used to visually examine the effect sizes for evidence of asymmetry (Sterne et al., 2011). In the absence of bias, effect sizes are expected to form a symmetrical funnel shape when they are scattered against the inverse of their standard error, with more precise studies appearing narrowly at the top of the plot and less precise studies scattering more widely at the bottom (precision increases with the inverse of the standard error). If studies are missing from one side of the plot and the funnel shape appears asymmetric, this could indicate the presence of publication bias. The visual inspection of the funnel plots indicated no clear evidence of asymmetry (see Appendix B). To test this statistically, Egger’s regression method was used (Egger, Smith, Schneider, & Minder, 1997). The results indicated no significant evidence for funnel plot asymmetry in either FFD (*t*(14) = 1.70, *p* = 0.11) or GD (*t*(17) = 1.33, *p* = 0.19). The effect sizes from Chinese studies were not analysed statistically for funnel plot asymmetry because such analyses are not recommended when there are fewer than 10 studies in the analysis (Sterne et al., 2011).

### Data Analysis

#### Meta-analysis

The goal of a meta-analysis is to pool together evidence from multiple studies in order to estimate the size of the underlying effect. Researchers can typically choose between a fixed-effect or a random-effects model. A fixed-effect model assumes that all studies are estimating the same true unknown effect size and any variability in the estimate can only be attributed to sampling error (Welton et al., 2012). On the other hand, a random-effects meta-analysis explicitly allows for variability between studies by assuming that each study has its own true effect that is generated from a normal distribution with some (unknown) mean and between-study variance. In this sense, the true effects of individual studies can be informally viewed as random samples from a normal distribution of effect sizes (Welton et al., 2012). The present meta-analysis used a random-effects model because it naturally allows for different sources of variability between studies (e.g., differences in stimuli materials or language of the study) that are common in psycholinguistic experiments.

The Bayesian random-effects model was defined as follows (Jäger et al., 2017; Welton et al., 2012). Let *T_i_* be the effect size of the phonological PB effect in the *i*th study, where *i* ranges from 1 to n (the number of studies included in the analysis). Let S_i^2 be the true variance of the effect size *T_i_*, which was estimated from the empirical variance calculated in Equations 1–2 above. Furthermore, let *θ_i_* be the true phonological PB effect in the *i*th study. Finally, let *θ* be the true, unknown phonological PB effect estimated by the model and τ^2^ the unknown between-study variance also estimated by the model. Therefore, the model was constructed as:

3 \begin{array}{l}
{T_i} \vert {\theta _i}, S_i^2 \sim Normal\left({{\theta_i}, S_i^2} \right), i = 1, 2, 3, \ldots {\rm{n}}\\
\qquad\qquad {\theta _i}\vert \theta, {\tau ^2} \sim Normal(\theta, {\tau ^2})\\
\qquad\qquad \theta \sim Uniform\left({-100, 100} \right)\\
\qquad\qquad \tau \sim Uniform(0, 100)
\end{array}

The present meta-analysis used the inverse-variance method, where the effect size estimate is weighted by the precision of individual studies (i.e., the inverse of the within-study variance of the sampling distribution). This means that more precise studies are given greater influence in the model. Because Bayesian inference is used, it is necessary to specify a prior distribution for the unknown parameters that are estimated by the model. In the present meta-analysis, the prior distributions were: θ ~ *Uniform* (–100, 100) and τ ~ *Uniform* (0, 100). These are vague or “uninformative” priors that have very little influence on the results. To confirm this, a sensitivity analysis was conducted with an alternative set of priors: θ ~ *Normal* (0,10^4^) and τ ~ *Normal* (0,10^4^) I[0,∞] (normal distribution truncated at 0). The sensitivity analysis showed that the choice of priors had no influence on the meta-analysis results or the conclusions from the analyses (see Supplementary file 1). We also explored in Supplementary file 1 how the results are influenced by more informative priors.

#### Meta-regression

While a random-effects meta-analysis explicitly allows for heterogeneity between studies, it does not help explain where this heterogeneity comes from (Welton et al., 2012). However, it is possible to use meta-regression to explicitly test how the effect size differs as a function of various study characteristics (e.g., whether the study used a homophone or a pseudo-homophone phonological preview). Meta-regression is an extension of the random-effects meta-analysis model in which a regression slope is added for the underlying effect of the covariate of interest. Meta-regression is therefore similar to the standard least-squares regression, but the difference is that it takes into account the precision of individual studies (Welton et al., 2012). The meta regression model was defined as follows (the added paramters are formatted in bold; Jäger et al., 2017; Welton et al., 2012):

4 \begin{array}{l}
{T_i}\vert {\theta_i}, S_i^2, {\boldsymbol \beta} \sim Normal\left({{\theta_i} + {\boldsymbol \beta} {{\boldsymbol x}_i}, S_i^2} \right),i = 1,2,3, \ldots {\rm{n}}\\
\qquad\qquad\quad {\theta_i}\vert \theta, {\tau^2} \sim Normal(\theta, {\tau ^2})\\
\qquad\qquad\quad \theta \sim Uniform (-100, 100)\\
\qquad\qquad\quad \tau \sim Uniform(0, 100)\\
\qquad\qquad\quad {\boldsymbol \beta} \sim \boldsymbol{Uniform}(-{\bf 100}, {\bf 100})
\end{array}

In this model, β is the regression slope for the effect of the covariate *x_i_*, *θ_i_* is the true phonological PB effect in the *i*th study after it has been adjusted for the covariate *x_i_*, and θ is the true unknown phonological PB effect (generating *θ_i_*), also adjusted for the covariate *x_i_*. All other parameters have the same interpretation as in Equation 3. Note that the addition of β also requires the specification of a prior for this parameter. For consistency purposes, a uniform prior was also used. Sum contrast coding was used for the covariate in all meta-regression models: Chinese vs. alphabetical/English studies (Chinese: 1; alphabetical/English –1), homophone vs. pseudo-homophone studies (homophone: 1; pseudo-homophone: –1). While meta-regression is a very useful tool, it should be noted that the results are only observational in nature (Thompson & Higgins, 2002) and should ideally be confirmed by future experiments that directly make these comparisons.

### Posterior sampling

Sampling of the posterior distribution was done with JAGS v. 4.30 (M. Plummer, 2003) in the R software v.3.5.1 (R Core Team, 2018). Ten Markov Chain Monte Carlo (MCMC) chains were run with 105 000 iterations each. The starting values were randomly generated, but checks were done to ensure that this did not influence the results. The first 5000 iterations were discarded as burn-in. Therefore, the summary of the posterior distribution was based on 1 million samples. Model convergence was assessed with Gelman and Rubin’s (1992) convergence diagnostic and with visual inspection of the parameter trace-plots. The evidence suggested that all models had converged. The effective sample size, or the size of the MCMC chains after adjusting them for auto-correlation (Kass, Carlin, Gelman, Neal, & Carlin, 1998), was always at least 10 000, as recommended by Kruschke (2015) (mean: 114 951; range: 13 190–437 168).

The results are reported as the estimate of the phonological PB effect in milliseconds and its 95% credible interval. Unlike confidence intervals, credible intervals (CrI) have the intuitive interpretation that they contain the true parameter of interest with a 95% probability because the values inside this interval correspond to 95% of the posterior distribution (see Morey, Hoekstra, Rouder, Lee, & Wagenmakers, 2016). Two probability measures are also reported for the meta-analysis results. The first one is the probability that the phonological PB is bigger than 0, given the data, i.e., p(Phon. PB > 0|Data). This is taken as evidence of how likely it is that the effect exists. The second one is the probability the phonological PB is bigger than 3 ms, given the data, i.e. p(Phon. PB > 3|Data). This is a more stringent criterion that quantifies the evidence in support of a small, positive effect. The cut-off of 3 ms was chosen because a potential phonological PB effect smaller than this number may be difficult to measure reliably. Because some experiments have used eye-tracker sampling frequency of 500 Hz, effects smaller than 2 ms may be too small to reliably capture with that resolution. Therefore, we chose a cut-off that is 1 ms larger than the lowest sampling resolution (i.e., 2 + 1 = 3 ms). For more information on Bayesian methods and their interpretation, readers are referred to Nicenboim and Vasishth (2016).

## Results

### Meta-analysis

**Figure 3:**
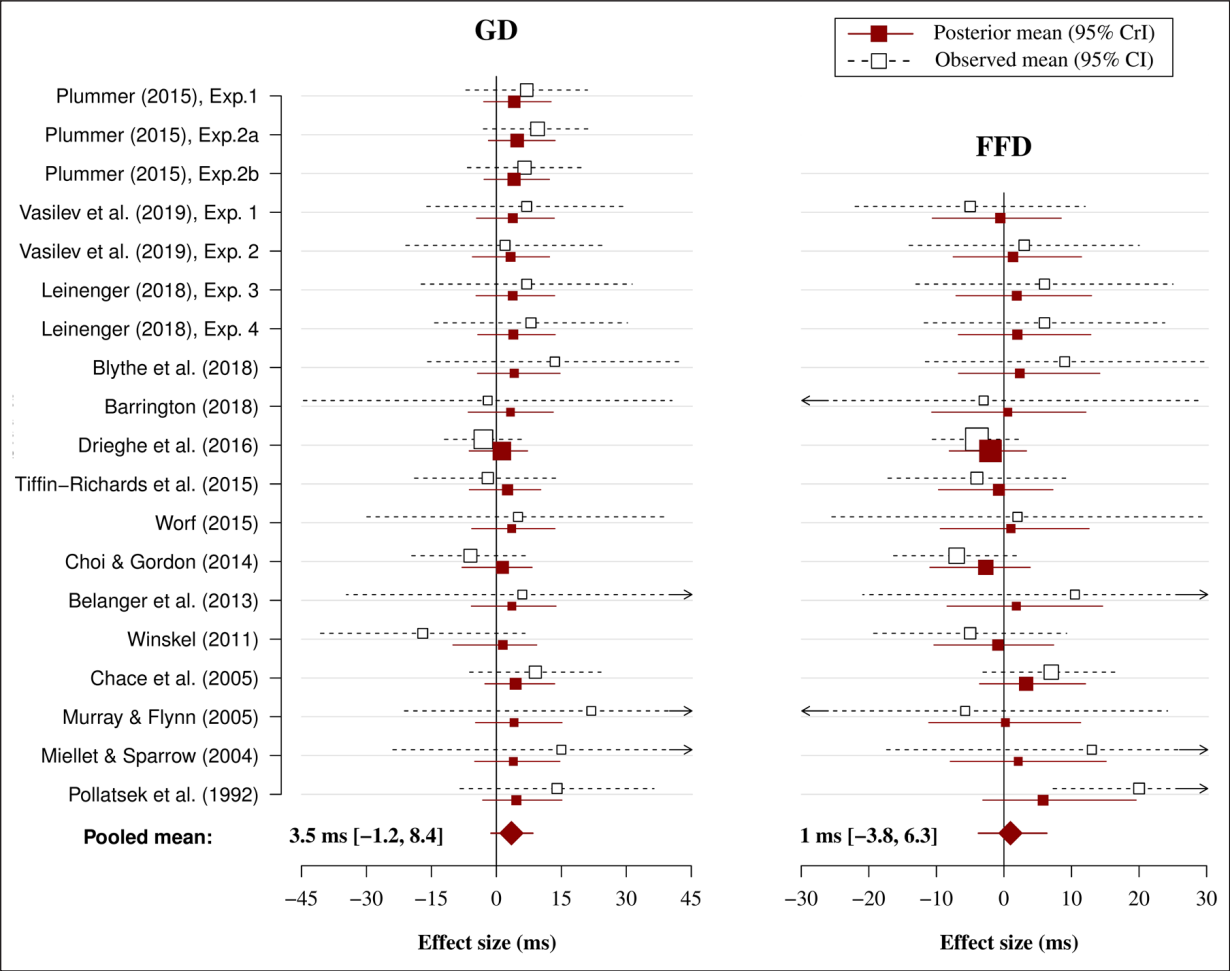
Forest plot of the alphabetical meta-analysis model for FFD and GD. Plotted are the observed study means (and 95% CI) reported in the original papers and the posterior means (and 95% CrI) estimated by the Bayesian meta-analysis model. The size of squares is proportional to the weight of each study in the analysis (i.e., the inverse of the within-study variance of the sampling distribution).

**Figure 4:**
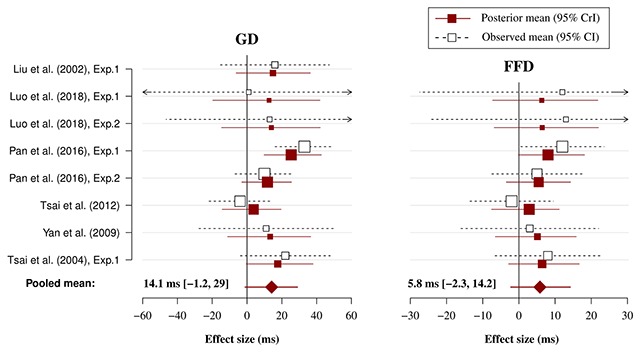
Forest plot of the Chinese meta-analysis model for FFD and GD. Plotted are the observed study means (and 95% CI) reported in the original papers and the posterior means (and 95% CrI) estimated by the Bayesian meta-analysis model. The size of squares is proportional to the weight of each study in the analysis (i.e., the inverse of the within-study variance of the sampling distribution).

The results from the meta-analysis are shown in Table 3. Additionally, the results are visualised in Figure 3 for alphabetical studies and in Figure 4 for Chinese studies. The estimated phonological PB effect measured in FFD was very small for both alphabetical (1 ms) and English-only studies (1.9 ms). This suggest that readers of alphabetical orthographies obtain little, if any, benefit from phonological previews during the first fixation of the target word. This conclusion was also supported by the finding that the probability of a true effect in FFD was only 73% for English studies. Interestingly, however, the phonological PB effect was much larger for Chinese studies in FFD (5.8 ms) and there was a 93% probability that the effect exists. Therefore, this suggests that it is very likely that Chinese readers benefit from a phonological preview during the first fixation of the target word.

**Table 3:** Posterior Effect Size Estimate of the Phonological Preview Benefit and 95% Credible Intervals from the Meta-Analysis Model.

Type of analysis	k	Mean ES (ms)	95% CrI	p(ES > 0|D)	p(ES > 3|D)	τ^2^	I^2^

**FFD**							
Alphabetical	16	1	[–3.7, 6.3]	0.64	0.20	22.4	5.0%
English-only	13	1.9	[–3.7, 8.3]	0.73	0.33	33.2	15.6%
Chinese	7	5.8	[–2.2, 14.2]	0.93	0.77	28.4	0.0%
**GD**							
Alphabetical	19	3.5	[–1.2, 8.4]	0.92	0.57	11.9	0.0%
English-only	16	4.5	[–0.4, 9.9]	0.96	0.72	12.9	0.0%
Chinese	8	14.1	[–1.1, 29.1]	0.96	0.93	173.9	26.2%

*Note*: FFD: first fixation duration. GD: gaze duration. k: number of studies included in the analysis. p(ES > 0|D): probability that the effect size is positive, given the data. p(ES > 3|D): probability that the effect is bigger than 3 ms, given the data. CrI: credible interval. τ^2^: estimated between-study variance. I^2^: percentage of variance that can be attributed to between-study heterogeneity.

The analysis of GD showed a modest phonological PB effect for both alphabetical (3.5 ms) and English-only studies (4.5 ms). The probability of a true effect was very high in both cases (≥93%), thus suggesting that it is very likely that readers of alphabetical orthographies benefit from phonological previews in GD. Interestingly, the phonological PB measured in GD was about 3 times as large for Chinese studies (14.1 ms) and the probability of a true effect was equally as high (96%). This suggests that Chinese readers are also very likely to benefit from phonological previews in GD. However, the uncertainty around this estimate was much larger. This was likely due, at least in part, to the smaller number of studies included in this analysis. However, the between-study variance (τ^2^) was also much larger compared to the other analyses, thus suggesting that the included studies differed between each other to a greater extent. The probability that the effects are larger than 3 ms, given the data, was more than 90% only for the Chinese effect in GD. However, the same probability for English in GD and Chinese in FFD was around 70%. This suggests that there was smaller probability that the effects are at least 3 ms in size.

A sensitivity analysis using the leave-one-out method was conducted to test the robustness of the meta-analysis results (see Greenhouse & Iyengar, 2009). In the leave-one-out method, the meta-analysis is repeated by omitting one different study each time. The results (presented in Supplementary file 1) indicated that the effect in GD for all languages and in FFD for Chinese studies was robust and not unduly influenced by any individual study. The effect in FFD for alphabetical and English studies was less robust, but the evidence for a phonological PB in this measure was not very strong to begin with.

### Meta-regression

Meta-regression analyses were used to test whether the phonological PB differs between alphabetical and Chinese studies, and between homophone and pseudo-homophone previews. The meta-regression slope from this analysis indicates the estimated mean difference between the two groups of studies after adjusting it for the precision of individual experiments. There was an 88% probability that the phonological PB is larger for Chinese compared to alphabetical orthographies in FFD (mean difference: b = 2.4 ms; 95% CrI: –1.8, 6.5 ms) and a 97% probability for such difference in GD (mean difference: b = 5.3 ms, 95% CrI: –0.1, 10.7 ms). Likewise, there was an 82% probability that the phonological PB effect is larger for Chinese compared to English studies in FFD (mean difference: b = 2.1 ms, 95% CrI: –2.5, 6.47 ms) and a 95% probability that it is also larger in GD (mean difference: b = 4.8 ms, 95% CrI: –0.83, 10.3 ms). Therefore, these results suggest that the size of the phonological PB is larger in Chinese compared to alphabetical orthographies.

A separate model indicated that the phonological PB was 1.57 ms larger for homophone compared to pseudo-homophone previews in FFD (95% CrI: –3.5, 6.6 ms) and 0.66 ms *smaller* in GD (95% CrI: –5.4, 4.2 ms). The probability of a true advantage for homophone previews was 74% for FFD and 38% for GD. Therefore, the results suggest that there was generally no difference in the amount of benefit that readers of alphabetical orthographies obtain from homophone and pseudo-homophone previews.

### Statistical Simulations of Missing Studies

While the statistical analyses indicated no evidence for the presence of publication bias, the survey of the unpublished literature nevertheless showed that there are additional unpublished studies for which no further information could be obtained. Although this does not in itself indicate the presence of bias, it does suggest that there is additional information that could not be accessed in the present meta-analysis. In light of this, statistical simulations were carried out using the survey findings to test the robustness of the meta-analysis results. Only the effect size in GD was analysed because it was generally larger.

**Figure 5:**
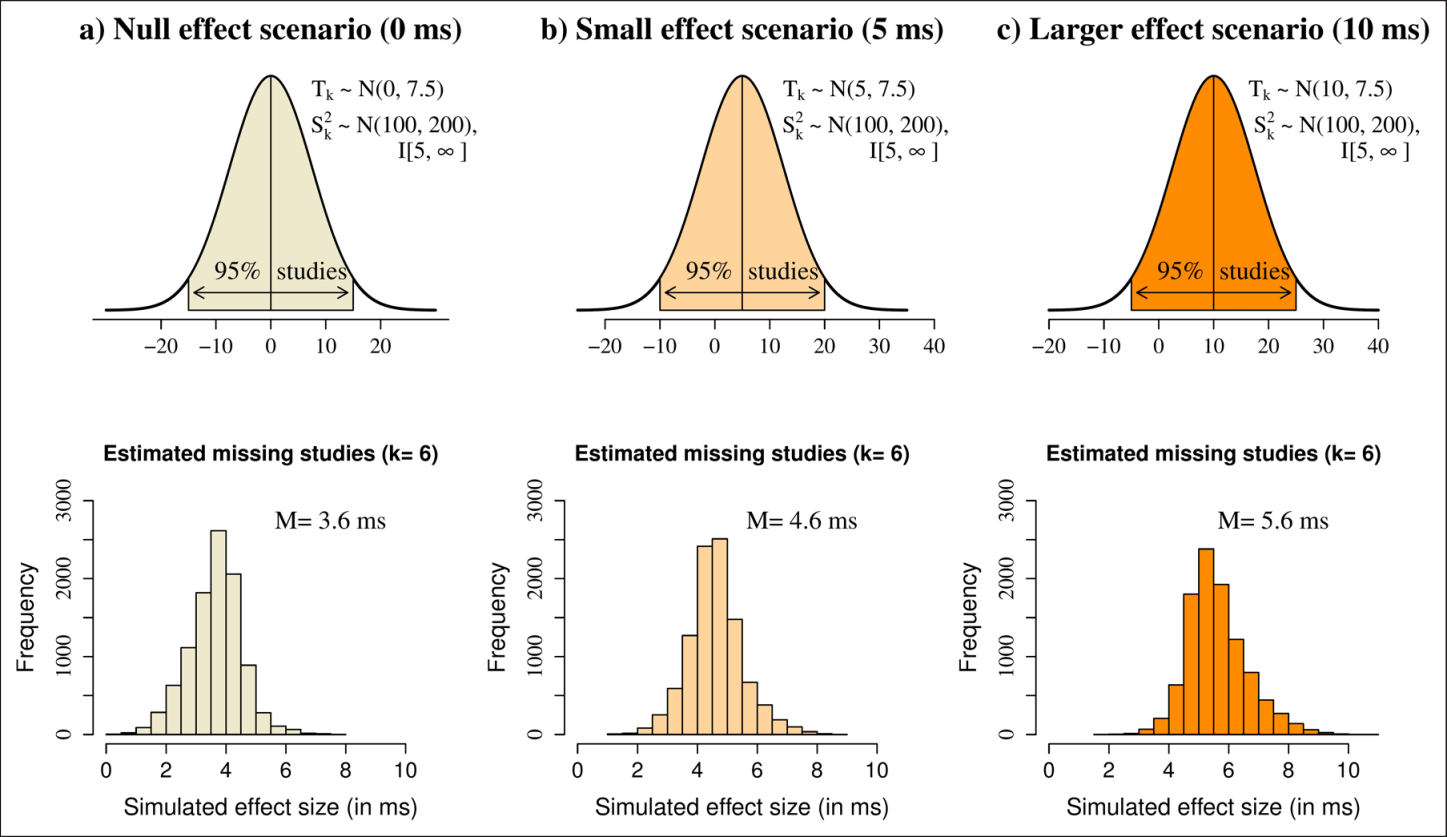
Statistical simulations with the estimated number of missing studies for the phonological PB effect in English (measured with gaze duration). The top row shows the three scenarios used to simulate the missing studies: a null effect **(a)**, a small effect **(b)** and a larger effect **(c)**. Each scenario represents a probability distribution from which the effect sizes of missing studies are assumed to come from. The second row shows the distribution of posterior effect sizes generated under each scenario with the estimated number of missing studies. The simulated effect sizes were generated by drawing a k number of missing studies from the respective distribution, adding them to the available studies from the paper, and repeating the meta-analysis model. All results are based on 10 000 Monte Carlo simulations.

Three different scenarios were used to simulate different types of missing data that may exist (see the top row of Figure 5 for an illustration). First, a “null effect” scenario assumed that all missing studies come from a normal distribution with a mean of 0 ms and a standard deviation of 7.5 ms. This is the most conservative scenario in which all missing studies are generated from a null effect and 95% of all studies fall between –15 and 15 ms. A second “small effect” scenario assumed that all missing studies come from a normal distribution with a mean of 5 ms and a standard deviation of 7.5 ms. In this scenario, the missing studies are generated by a small (5 ms) effect and 95% of all studies lie between –10 and 20 ms. A final “larger effect” scenario assumed that all missing studies come from a normal distribution with a mean of 10 ms and a standard deviation of 7.5 ms. In this case, the studies are generated by a larger (10 ms) effect and 95% of all studies lie between –5 and 25 ms. The variance of effect sizes was kept constant across the three scenarios; it was sampled from a normal distribution with a mean of 100 ms and a standard deviation of 200 ms that was truncated at 5 ms as its lower bound (the upper bound was +∞). The distribution was truncated at 5 rather than 0 ms because variances very close to 0 are highly unlikely given the typical precision of studies on this topic. This distribution was used because it is a good approximation of the variability of parafoveal preview effect sizes typically observed in the literature (see Vasilev & Angele, 2017).

The general method for the simulations was as follows. For each of the *k* missing studies, an effect size *T_k_* and a variance of this effect size S_k^2 were randomly drawn from the respective probability distributions. S_k^2 was always drawn from the same distribution: S_k^2 \sim {\rm{Normal}}(100, 200){\rm{I}}[5,\infty]. The distribution from which *T_k_* was drawn differed based on the simulation scenario (*T_k_* ~ Normal(0,7.5) [“null effect”]; *T_k_* ~ Normal(5,7.5) [“small effect”]; *T_k_* ~ Normal(10,7.5) [“larger effect”]). The *k* effect sizes and their variances were then added to the available data and the meta-analysis model was fit. This procedure was repeated 10 000 times for each scenario, thus yielding 10 000 simulated effect size estimates of the phonological PB. The simulations were done once with the number of known missing studies (*k* = 4; see Figure 2b) and once with the estimated missing studies. The estimated percentage of missing studies was calculated by dividing the number of known missing studies by the total number of researchers who have done work on this topic (4/13 = 30.7%). The number 4 was taken because one researcher indicated that they had one unpublished study and three more researchers said that they had unpublished studies, but did not indicate how many. Therefore, the number of estimated missing studies was *k* = 6 for alphabetical orthographies, *k* = 5 for English and *k* = 2 for Chinese. It should be noted that these simulations assume that all researchers who failed to provide information about their unpublished studies in the survey have only one relevant unpublished experiment. This represents the lowest possible bound for how many unpublished studies exist.

The results are illustrated in Figure 5 for English studies and the results for all other analyses are shown in Table 4. The phonological PB effect measured in GD was generally robust to the missing studies for all languages. The smallest effect size for alphabetical orthographies remained positive even in the most conservative “null effect” scenario. However, the average effect in the simulations was somewhat smaller in this scenario, thus suggesting that the true phonological PB effect for alphabetical studies may be even smaller if all or most of the missing studies happen to be null results. A similar result was obtained for English-only studies, but the difference was that the effect was about 1 ms larger than that of alphabetical studies. There was more variability in the simulated effect size for Chinese studies, which was likely due to the smaller number of available experiments for that language. Nevertheless, the effect size was always at least 9 ms on average. In summary, the simulations revealed that the phonological PB is robust to the missing studies, but the actual size of the effect may be slightly smaller if most of them are null results.

**Table 4:** Results from 10 000 Monte Carlo Simulations of the Phonological PB Effect in Gaze Duration with Known and Estimated Number of Missing Studies (Standard Deviations in Parentheses).

Simulation type	k	ES (available data)	Mean ES from 10 000 Monte Carlo simulations		

			Null effect	Small effect	Larger effect

**Known missing**					
Alphabetical	4	3.5	2.97 (0.73)	3.71 (0.76)	4.46 (0.85)
English-only	4	4.5	3.77 (0.81)	4.59 (0.83)	5.46 (0.94)
Chinese	4	14.1	8.98 (1.67)	10.81 (1.62)	12.69 (1.59)
**Estimated missing**					
Alphabetical	6	3.5	2.76 (0.85)	3.78 (0.89)	4.86 (0.98)
English-only	5	4.5	3.65 (0.88)	4.61 (0.91)	5.62 (1)
Chinese	2	14.1	11.06 (1.44)	12.13 (1.4)	13.29 (1.35)

*Note*: k: Number of simulated missing studies. ES: phonological PB effect size (in gaze durations).

### Statistical Power Simulations

The meta-analysis revealed that there is a high probability that the phonological PB in GD is bigger than 0 (see Table 3). Therefore, one important question is to determine the number of subjects and items needed to reliably detect the effect (see Brysbaert, 2019; Brysbaert & Stevens, 2018). To do that, statistical power simulations were used where Linear Mixed Models (LMMs) were fit using simulated, but realistic data. This was done using the “lme4” R package v. 1.1-18-1 (Bates, Machler, Bolker, & Walker, 2014). In these models, GD was the outcome variable and experimental condition (phonological vs. control preview) was the only predictor variable. Random intercepts, as well as random slopes for experimental condition were added for both participants and items (Baayen, Davidson, & Bates, 2008; Barr, Levy, Scheepers, & Tily, 2013). The results were considered statistically significant if the *p*-values were ≤0.05. The simulations were carried out using the “simr” R package v.1.0.5 (Green & Macleod, 2016). The variance-covariance matrix of the random effects and the residual variance were estimated using the data from Leinenger (2018), Experiment 4 for English and Yan et al. (2009) for Chinese studies. Both datasets have a reasonable number of subjects and items; therefore, it was possible to simulate realistic data that can capture the by-subject and by-item variability that is present in the effect.

In the power simulations, the size of the phonological PB effect in GD was kept constant (4.5 ms for English and 14 ms for Chinese) while the number of statistical subjects and items was being manipulated. For each subject/item number permutation, 200 simulated LMMs were run and the phonological PB effect was calculated. The statistical power of the simulation was defined as the number of LMM models that showed a significant phonological PB effect divided by the total number of models run (e.g., 100 significant models out of 200 would yield a statistical power of 0.5). If more than 5% of the models in a given simulation yielded a warning or a convergence failure, the simulation was repeated until the proportion of such models was below 5%. If this did not happen within 5 attempts, the random slopes for experimental condition were removed to help the model converge (this occurred only 0.52% of the time).

**Figure 6:**
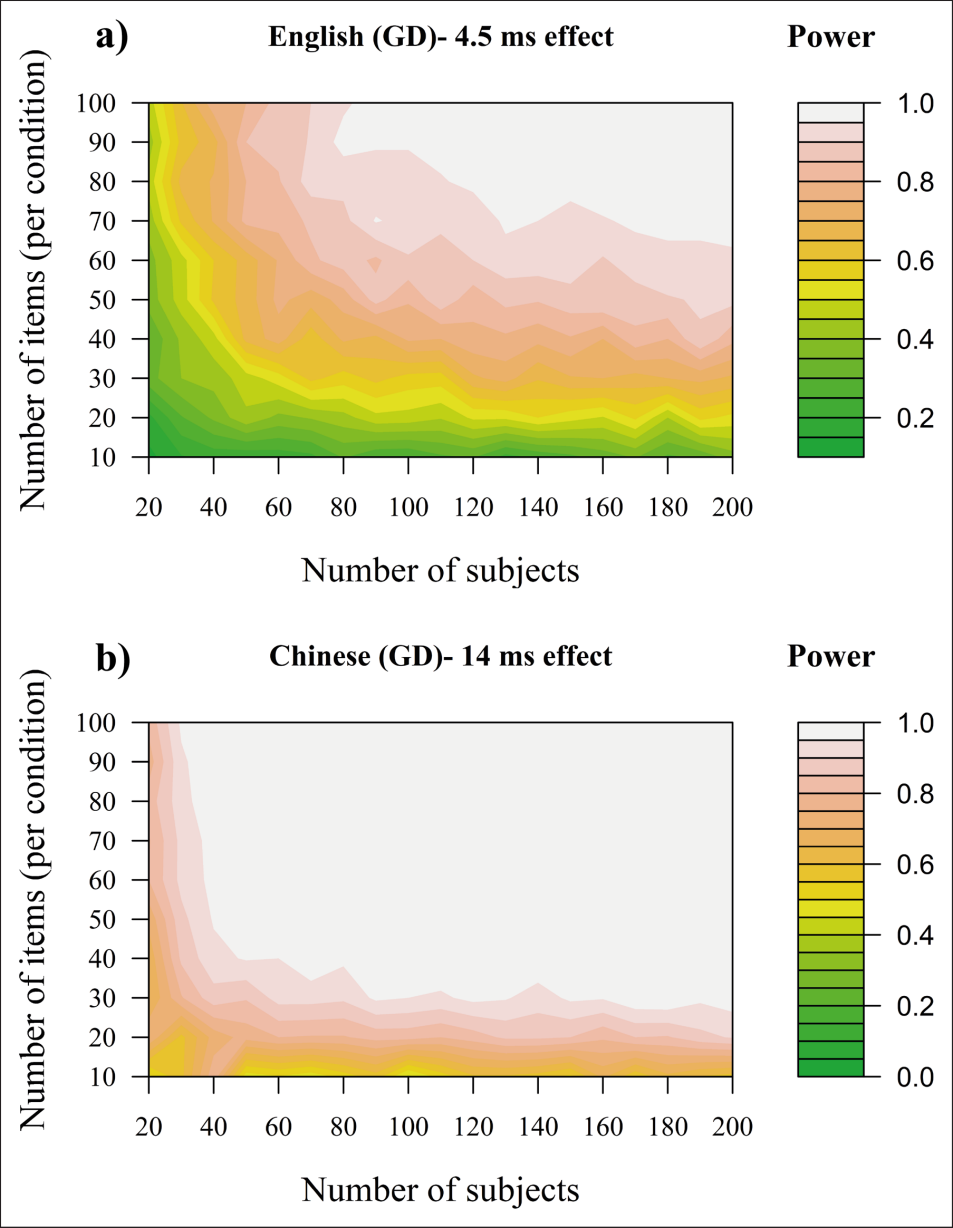
Number of subjects and items needed to achieve different levels of statistical power to detect the phonological PB in gaze durations for English (panel a) and Chinese (panel b). Statistical power is based on LMMs with a “maximum” random effects structure (Barr et al., 2013). Warmer colours indicate more desirable levels of statistical power.

The results are presented in Figure 6. For English, the effect could be detected with 80% power with at least 90 subjects and 50 items per condition. This suggests that, while the effect very likely reflects a true difference in the population, only high-precision experiments may be able to reliably capture it. Not surprisingly, the larger effect in Chinese was easier to detect and only 40 subjects and 30 items per condition were needed to achieve 80% statistical power.

## Discussion

The aim of the present study was to synthesize the available evidence on the phonological PB effect in order to estimate its size and the probability that it exists. Overall, the present results are broadly consistent with the notion that readers can process phonological information parafoveally. However, there are also a few important caveats to this conclusion. First, readers of alphabetical orthographies showed little if any evidence of a phonological PB effect in first fixation duration on the target word. Second, while the probability that the effect exists in gaze duration was very high (>92%), the magnitude of the effect was modest in size (4.5 ms for English studies). This suggests that the size of the phonological PB effect in alphabetical orthographies may be smaller than what has been sometimes assumed. The stronger effect in gaze durations could indicate that phonological codes are mostly integrated later on in the reading process when the target word is re-fixated. However, as the present results are only observational in nature, future experimental work is required to test this directly. Third, the phonological PB in alphabetical orthographies did not differ between homophone and pseudo-homophone previews, which suggests that the modest benefit is indeed related to the activation of phonological codes in the parafovea. Finally, the results also suggested that the phonological PB effect is *larger* in Chinese compared to alphabetical orthographies. This last result may be surprising as the opposite difference has sometimes been assumed (e.g., Tsang & Chen, 2012).

The larger effect size in Chinese should not be interpreted as reflecting cross-linguistic differences that favour greater phonological processing in this language for at least two reasons. First, because Chinese is an unspaced language, information is more densely packed and the parafoveal word will be generally closer to the point of fixation compared to alphabetical orthographies. As a result, there is a smaller loss of visual acuity in the parafovea, which may lead to more efficient parafoveal processing in general. In fact, previous research has shown that the classical PB effect (valid vs. invalid preview difference) is larger for Chinese compared to alphabetical orthographies in GD (Vasilev & Angele, 2017). In this sense, it is likely that at least some of the advantage for Chinese studies may originate from the more favourable viewing conditions.

Second, the two types of studies also had different baselines- while Chinese experiments used an unrelated-word control, alphabetical studies used an orthographically-related (non)word control. Therefore, the observed difference can also likely be explained by the different baselines. This is not necessarily a limitation of the present analysis as the choice of baseline is inherent to linguistic differences between these languages. Chinese has a deep orthography where phonology and orthography do not have the same overlap as most alphabetical languages. As a result, researchers have typically used an unrelated-word baseline (e.g., see Yan et al., 2009), presumably because it is easier to create phonological previews that have little orthographical overlap with the target. In this sense, the difference in baselines must be considered in any comparisons between the two types of languages (regardless of whether they are based on the present results or not). However, the advantage of the present study is that it directly quantifies this difference and makes it possible to derive numeric predictions for future research.

It is also worth noting that, while the phonological PB in English has often been considered as the benchmark against which to evaluate the effect in other languages, this does not necessarily mean that the effect is most robust in that language. In fact, it has often been noted that it is difficult to separate the phonological from the orthographical effect in English due to the overlap between orthography and phonology (e.g., Choi & Gordon, 2014; Jouravlev & Jared, 2018; Winskel, 2011). Even though English lacks the more transparent spelling-to-sound mapping of languages such as Finnish or Italian, it still has a considerable correspondence between letters and phonemes. This inevitably means that the phonological preview will share not only phonology, but also orthography with the target, and that a good orthographic control condition needs to be used to isolate the phonological contribution to the effect. Additionally, the orthographic control condition usually shares at least one phoneme with the target (typically the first one), which should lead to some phonological activation of the control which makes the detection of the effect more difficult.

In contrast, because Chinese uses a non-alphabetical orthography, it does not have the same spelling-to-sound mapping as English. In Chinese, characters represent different morphemes and these morphemes cannot be linearly combined to form the pronunciation of words (Luo et al., 2018). This means that phonology is not reliant on orthography in the same way that it is in English, and readers have to rely on the phonetical radical of compound characters to get an indication of the character’s pronunciation (Wengang, 1991). Therefore, the controls in Chinese studies should not lead to the activation of phonological codes present in the targets, which may make it easier to measure the phonological PB effect than with alphabetic orthographies such as English.

### Implications for the Strong versus Weak Phonological Viewpoints

As noted above, there are two competing viewpoints on the role of phonology during visual word recognition. According to the strong phonological view (Frost, 1998), the phonological code is a mandatory component in visual word recognition. That is, phonology mediates the activation between letter/graphemes and the orthographic word form. On the other side of the debate is the weak phonological view (e.g., Rastle & Brysbaert, 2006), which posits that the phonological code is not mandatory as the orthographic code can be accessed directly from incoming letter level activation.

Masked phonological priming in visual word recognition has long been considered as compelling evidence for the strong phonological view, and the effect has been observed using several different priming paradigms. The relevance of the present research is that it provides additional evidence in favour of the strong phonological view. As the results show, there is a modest but reliable phonological PB effect. It is important to emphasize that the veracity of the strong phonological view does not depend on the size of the phonological PB effect, but rather on its existence. Indeed, given the relatively small phonological priming effects in visual word recognition (Rastle & Brysbaert, 2006), a small phonological PB effect is also to be expected, particularly when syntactic and semantic constraints on word recognition come into play during text reading. The fact that there is a phonological PB effect at all adds further support to the view that word recognition and reading require phonological activation.

### Implications for Future Studies

The present results also have important implications for future studies investigating the parafoveal processing of phonology. For example, the power simulations indicated that a high number of subjects and items may be required to achieve desirable levels of statistical power, at least for alphabetical orthographies such as English. Additionally, future studies investigating how the effect may be modulated by different factors (e.g., participant population, individual differences) should consider the general size of the effect, as interaction terms may be even harder to detect. This may be particularly important when studying rare populations, such as deaf or dyslexic readers, where large sample sizes may be difficult to achieve in practice. In such cases, it may be desirable to seek converging sources of evidence- for example, from phonological priming in foveal vision where the effects may be larger.

While the present results are consistent with the existence of a small phonological PB effect, they do not tell us whether larger effects may be observed under some conditions. For example, one interesting recent hypothesis is that stronger phonological PB effects may be observed in oral compared to silent reading due to the need for articulation in the former reading mode (Pan et al., 2016). While this hypothesis has only been tested in Chinese so far, a similar effect would also be predicted in alphabetical orthographies such as English. Additionally, the phonological PB effect may be modulated by individual differences such as reading skill level. This possibility was first suggested by Chace et al. (2005), who reported that only skilled adult readers were able to process phonology parafoveally. However, the way that participants were divided into “skilled” and “less skilled” readers in their study was somewhat arbitrary and it may be beneficial to replicate their results independently. Nevertheless, as there is a growing number of studies indicating that the amount of parafoveal processing may depend on reading skill level (Veldre & Andrews, 2014, 2015a, 2015b, 2016b), this remains an interesting question to study in the future.

Because the present study had access only to the experiment-level effect sizes, it was not possible to examine whether the phonological preview benefit may be modulated by different linguistic properties of the target words, such as their lexical frequency (Risse & Kliegl, 2012, 2014; Risse & Seelig, 2019; Schotter & Leinenger, 2016; Schotter, Leinenger, & von der Malsburg, 2017; Schotter, von der Malsburg, & Leinenger, 2019) or plausibility (Schotter & Jia, 2016; Veldre & Andrews, 2016a). Therefore, this remains a task for future studies. To facilitate such analyses, we encourage researchers to routinely make their data openly available (Gilmore, Kennedy, & Adolph, 2018; Martone, Garcia-Castro, & Vandenbos, 2018; Vanpaemel, Vermorgen, Deriemaecker, & Storms, 2015).

### Implications for Computational Models of Reading

Parafoveal processing is often thought to be a key part of skilled adult reading (e.g., Reichle & Reingold, 2013) and has played an important role in the development of computational models of eye-movement control, such as E-Z Reader (Reichle et al., 1998), SWIFT (Engbert et al., 2005), and more recently OB1 (Snell et al., 2018). Nevertheless, even though all three models allow for parafoveal processing, neither E-Z Reader nor SWIFT explicitly implement how different types of linguistic information, such as phonology, orthography, or semantics may be processed parafoveally. Even the more recent OB1 model (Snell et al., 2018), which includes open-letter bigrams that are activated by the visual input and subsequently excite word-level nodes, would only be capable of capturing linguistic effects to the extent that they correlate with the open-letter bigram codes. Therefore, currently no models of eye-movement control during reading explicitly explain how phonology is processed parafoveally, even though such accounts do exist in (foveal) single-word recognition (e.g., Coltheart et al., 2001; Perry et al., 2007). Rather, because the classical PB is often thought to be related to the activation of orthographic and phonological codes (Rayner et al., 2003), any model that can account for this effect is also by association consistent with the existence of the phonological PB.

Nevertheless, future models of reading may explicitly add components for the parafoveal processing of phonology, much in the same spirit as OB1 (Snell et al., 2018) has done for orthography. The present research places important constraints on future models by suggesting that only a limited amount of phonological processing may occur parafoveally, at least in alphabetical orthographies such as English. If the classical PB is indeed the result of orthographic and phonological activation (Rayner et al., 2003), then the present results suggest that the contribution which is unique to phonology is modest.

The limited size of the phonological PB effect is not necessarily surprising as the effect is not very large in single word recognition studies. For example, Rastle and Brysbaert (2006) found that phonological masked priming effects in visual word recognition are about 10–13 ms in size. Because the phonological prime in such studies is viewed foveally, the results may represent a “theoretical maximum” as to how much phonological processing can occur. In contrast, parafoveal processing necessarily incurs costs due to the decrease in visual acuity, and as a result, the effects are likely to be smaller. This is consistent with computational models of reading in which the amount of processing is modulated by the eccentricity of the parafoveal word from the point of fixation. This is perhaps more obvious in models of spatially distributed processing where either the visual input (OB1; Snell et al., 2018) or the lexical processing rate of words (SWIFT; Engbert et al., 2005) are a function of the eccentricity from the fixation point. However, even sequential-attention models such as E-Z Reader assume that the time to complete the first stage of lexical processing (L1) is modulated by the mean absolute distance between each of the letters in the word and the gaze position (Schotter, Reichle, & Rayner, 2014). Therefore, existing models are consistent with the notion that phonological processing should be smaller in parafoveal compared to foveal vision. In this sense, given the loss of visual acuity in the parafovea, the present results suggest that phonological PB effects may be smaller in practice than the effects observed in foveal phonological priming studies (see Rastle & Brysbaert, 2006).

## Conclusion

It has commonly been accepted that readers are able to integrate phonological codes across saccades, but no systematic attempt has been made to quantify the evidence in support of this conclusion. The results of the present meta-analysis showed that readers can indeed process phonological information parafoveally and later use it to aid foveal word recognition. Simulations revealed that the results are relatively robust to missing studies, although the effects may be 19–22% smaller if all missing studies found a null effect. Nevertheless, the magnitude of the effects was modest, particularly for alphabetical orthographies such as English. Therefore, the present results suggest that the unique phonological processing contribution to the classical PB effect is small, and the rest is likely due to orthographic and other influences.

Interestingly, Chinese readers showed a stronger phonological PB effect, although this may occur in part due to differences in viewing conditions that make Chinese more conducive to parafoveal processing in general (see Vasilev & Angele, 2017). The small phonological PB effect in English is also consistent with the results from phonological masked priming studies in single word recognition (Rastle & Brysbaert, 2006). Because there is a loss of visual acuity in the parafovea, readers are likely better at processing phonology foveally than parafoveally, which explains why phonological PB effects may be small. However, the relatively larger effects in Chinese compared to English may also be related to the differences in controls used in the two languages. With alphabetic languages like English, orthographic control previews also overlap phonologically with the target words to varying extents. Since evidence of phonological PB involves a comparison to this control condition, any shared phonology between the control and the target will dilute the measurement of this effect. In Chinese, the control previews do not share phonological codes with the targets and therefore the measurement of the effect will not be as diluted as it is in English. More research is needed to understand if there are conditions that may foster greater phonological processing during text reading.

## Data Accessibility Statement

The data files and analysis scripts used in this study are openly available at: https://doi.org/10.17605/OSF.IO/7349S.

## Additional File

The additional file for this article can be found as follows:

10.5334/joc.87.s1 Supplementary file 1. Additional analyses from the paper.

## Figures and Tables

**Figure B1:**
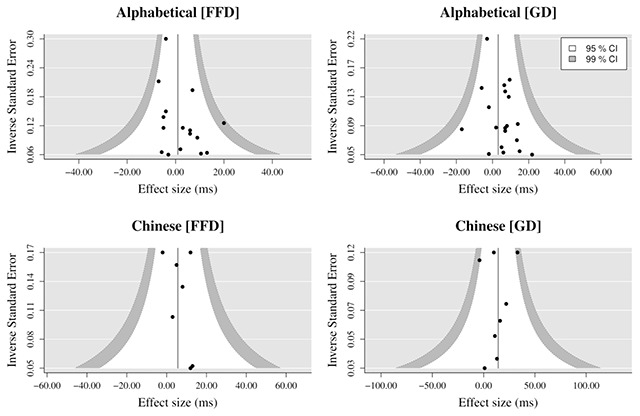
Funnel plots of the studies included in the meta-analysis. In the absence of bias, more precise studies are expected to appear narrowly at the top of the plot, whereas less precise studies are expected to scatter more widely at the bottom of the plot.

## Appendix A

Inclusion criteria for the meta-analysis (adapted from Vasilev & Angele, 2017):

- The study used Rayner’s (1975) boundary paradigm to manipulate the parafoveal preview of a target word in a sentence.
- The study had a phonological preview condition and a suitable control preview condition (orthographic control for alphabetical studies and an unrelated-word control for Chinese studies). Phonological preview was defined as a word/nonword that was homophonous/pseudo-homophonous with the target word.
- Reading was silent.
- Participants were native speakers in the language that they were reading and only a single language was used in the experiment (i.e., no cross-language priming).
- The population was healthy, skilled adult readers (typically university students). Participants had to be at least 18 years on average to be considered adults.
- The study reported descriptive statistics for first fixation duration, gaze duration, or both.
- Any additional manipulations other than parafoveal preview of the target word (if present) were not too disruptive to the reading process. Such manipulations were averaged out.
- Researchers took reasonable measures to ensure good eye-tracking data quality (e.g., calibration, drift check/correction, head-/chin-rest).
