# Supplementary material for: Do Readers Integrate Phonological Codes Across Saccades? A Bayesian Meta-Analysis and a Survey of the Unpublished Literature
Source: J Cogn. 2019 Oct 4;2(1):43. doi: 10.5334/joc.87 (PMC6838770; doi:10.5334/joc.87)
Supplement: Supplementary file 1. — Additional analyses from the paper. [file joc-2-1-87-s1.pdf]

Supplementary file 1

Sensitivity Analysis Results (Leave-one-out Method)

Table S1

Robustness Check of the Meta-Analysis Results Using the Leave-one-out Method

Analysis	ES reported in paper (ms)	Leave-one-out results			
		Mean ES	SD of ES	Min ES	Max ES
**FFD**					
Alphabetical	1	1.01	0.66	-0.56	2.1
English only	1.9	1.95	0.87	0.037	3.36
Chinese	5.8	5.87	1.19	4.05	8.05
**GD**					
Alphabetical	3.5	3.52	0.49	2.81	4.81
English only	4.5	4.6	0.59	3.95	6.31
Chinese	14.1	14.1	2.71	8.97	18.85

Note: ES: effect size.

Sensitivity Analysis with Different Priors

To confirm that the meta-analysis results are not influenced by the choice of priors, sensitivity analyses with another set of uninformative priors were conducted: $\theta \sim \textit{Normal}\ (0,10^4)$ and $\tau \sim \textit{Normal}\ (0,10^4)\ I(0,\infty)$. The results (presented in Figure S1) showed that the choice of uninformative priors did not influence the findings from the meta-analysis.

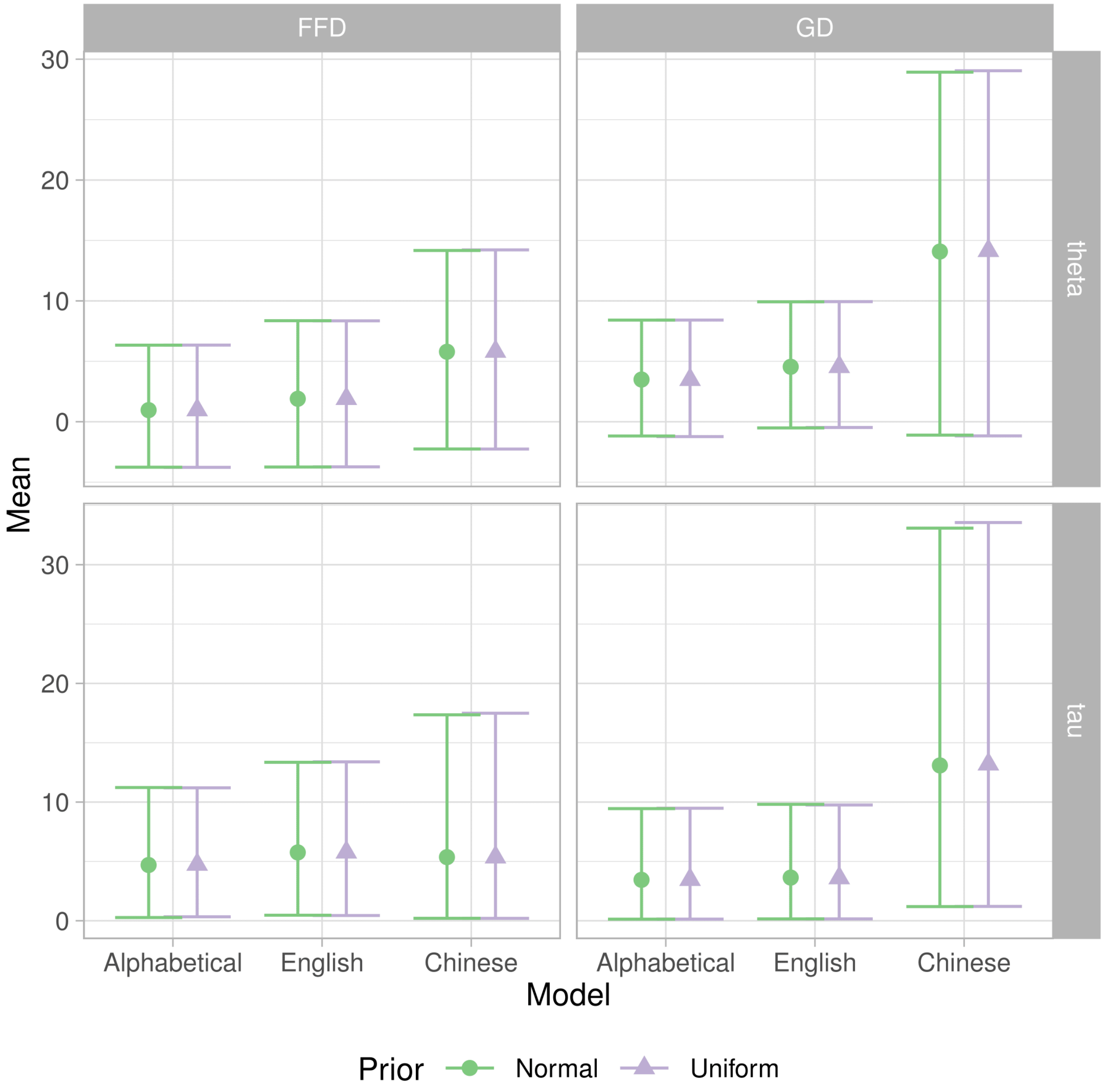

Figure S1. Sensitivity analysis of the meta-analysis results using a different set of priors. Plotted are the Uniform distribution priors used in the results from the paper [θ ~ *Uniform* (-100, 100) and τ ~ *Uniform* (0, 100)] and the alternative Normal distribution priors used in the sensitivity analysis [θ ~ *Normal* $(0,10^4)$ and τ ~ *Normal* $(0,10^4)$ I(0,∞)]. The sensitivity analysis indicated that the choice of priors did not influence the meta-analysis results or its conclusions.

However, because the priors used in Figure S1 were uninformative, one interesting question is under what circumstances the choice of priors may start to influence the results. We examined this question by using Normal distribution priors that differed in how informative they were. One important factor that influences the "informativeness" of Normal priors is their standard deviation. When the standard deviation is sufficiently large (e.g. 10^4, as in the analysis above), normal distributions become "diffuse" or non-informative over the range of plausible values where the phonological preview benefit may lie [-100, 100 ms]. Therefore, Normal priors become more "informative" when their standard deviation gets increasingly smaller because the probability density is constrained over a much smaller range of values. For example, in a Normal distribution with a mean of 0 ms and a standard deviation of 10 ms, 95% of all values will lie between -20 and 20 ms. When Normal priors are informative, their mean also starts to matter because it determines where most of the probability density is centred. Therefore, we also varied the mean of the priors.

The priors used in the simulation are shown in Table S2. In this analysis, standard deviations of 10000, 1000, 100, and 10 ms were used (this corresponds to priors that become increasingly more informative). Additionally, the mean of the Normal priors was either 0 ms (as in the analysis in Figure S1) or 10 ms. Of course, individual researchers may choose different values depending on their expectations of the size of the effect, but the purpose of the present analysis was simply to test how the results may change with a range of realistic priors.

Table S2

Additional Prior Distributions Used to Assess how the Results are Influenced by More Informative Priors

Model	Mean of prior distribution	SD of prior distribution	Used priors (notation)	
			θ (theta)	τ (tau)
**M1**	**0**	**10000**	**θ ~ *Normal* (0, 10000)**	**τ ~ *Normal* (0, 10000) I(0, ∞)**
M2	0	1000	θ ~ *Normal* (0, 1000)	τ ~ *Normal* (0, 1000) I(0, ∞)
M3	0	100	θ ~ *Normal* (0, 100)	τ ~ *Normal* (0, 100) I(0, ∞)
M4	0	10	θ ~ *Normal* (0, 10)	τ ~ *Normal* (0, 10) I(0, ∞)
M5	10	10000	θ ~ *Normal* (10, 10000)	τ ~ *Normal* (10, 10000) I(0, ∞)
M6	10	1000	θ ~ *Normal* (10, 1000)	τ ~ *Normal* (10, 1000) I(0, ∞)
M7	10	100	θ ~ *Normal* (10, 100)	τ ~ *Normal* (10, 100) I(0, ∞)
M8	10	10	θ ~ *Normal* (10, 10)	τ ~ *Normal* (10, 10) I(0, ∞)

Note: Model M1 (formatted in bold) was used in the sensitivity analysis in Figure S1 and yields effectively the same results as the analyses reported in the main paper.

The results from the sensitivity analysis with more informative priors are presented in Figure S2. There are a few important trends to note. First, as the priors become more informative (i.e., with decreasing SD), the posterior distribution becomes steeper and narrower. This occurs because there is a stronger a priori expectation of where the effect should lie. Nevertheless, as Figure S2 shows, most of the probability mass still falls to the right of 0, which is consistent with the existence of the phonological preview benefit effect. Second, when the mean of the prior changes from 0 to 10 ms, the phonological preview benefit shifts towards more positive values. This occurs because such priors assume that the effect is positive, so the posterior distribution is

also shifted towards more positive values. Therefore, if researchers have stronger prior expectations that the effect will be positive, they will generally obtain larger effect sizes.

Finally, the analyses also show that the model with Chinese studies is most strongly influenced by the choice of priors. This is not surprising as there are fewer Chinese studies compared to alphabetical studies and the priors exert a stronger influence on the posterior distribution when there are fewer observations. This occurs because the posterior distribution is proportional to the product of the prior distribution and the likelihood of the data (see Lynch, 2007). Therefore, in the absence of a lot of data, the priors will tend to have a stronger influence on the results. Even so, the results from Chinese studies still don’t change dramatically as the effect is mostly positive and covers a similar range of values. In summary, using more informative priors will lead to a greater change in the results. However, if reasonable informative priors are used, the main conclusions from the analysis will not change.

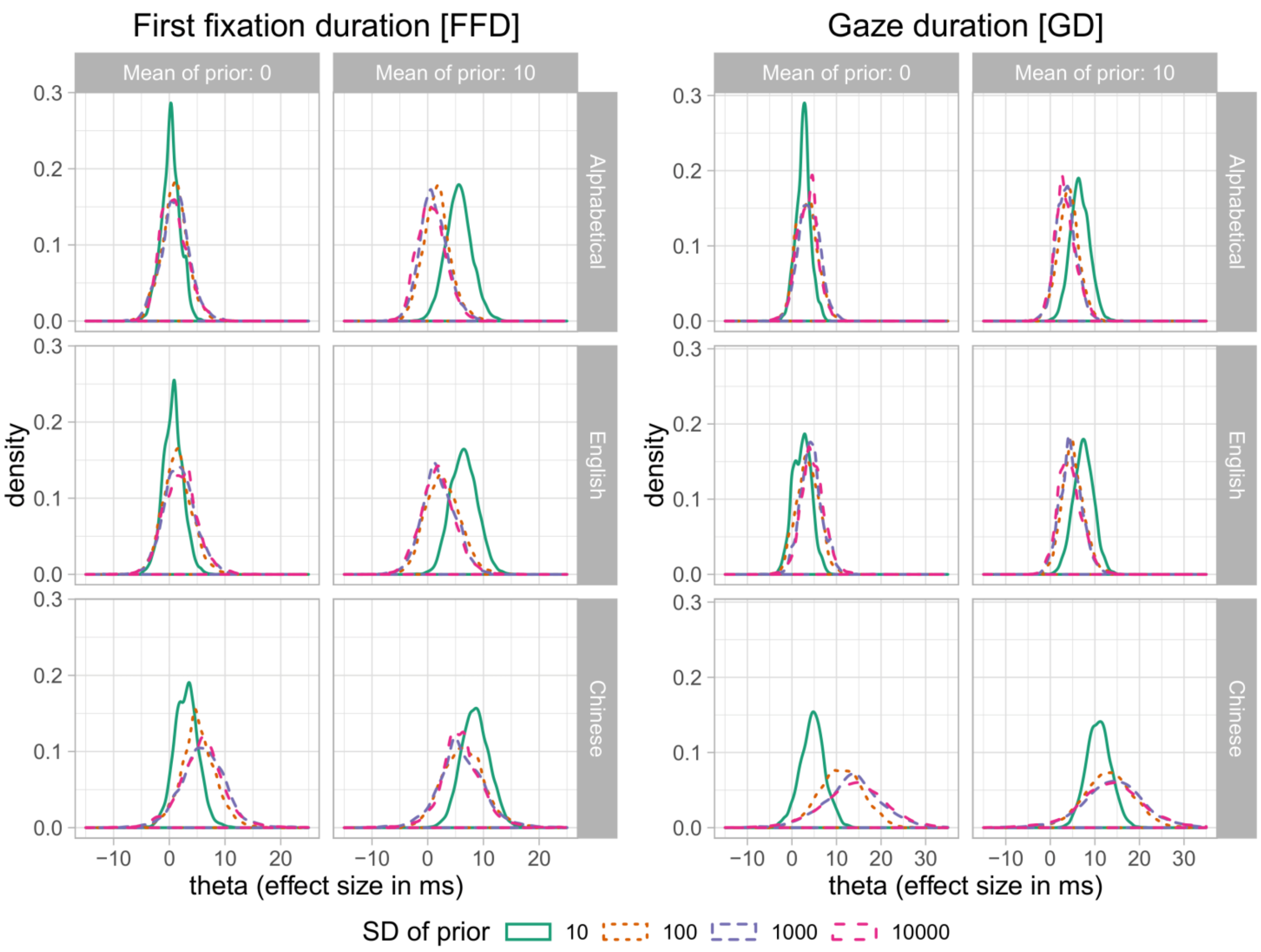

Figure S2. Sensitivity analysis with more informative prior distributions (see Table S2 for details). Plotted are the posterior probability distributions of the phonological preview benefit effect (theta) for the different types of priors. The Normal prior distributions differed with respect to how wide they were (standard deviations of 10, 100, 1000, or 10000 ms) and whether the normal distributions were centred at 0 or 10 ms. Normal priors become more informative when their standard deviation is smaller because they constrain the range of expected values. The model with a mean of 0 ms and standard deviation of 10000 ms yields results that are effectively the same as those reported in the main paper.

References

Lynch, S. M. (2007). *Introduction to applied Bayesian statistics and estimation for social scientists*. Berlin: Springer.
